# Applying the Partial Order Continual Reassessment Method to High‐Dimensional Treatment Combinations

**DOI:** 10.1002/sim.70345

**Published:** 2026-02-27

**Authors:** Weishi Chen, Li Liu, Nolan A. Wages, Pavel Mozgunov

**Affiliations:** ^1^ MRC Biostatistics Unit University of Cambridge Cambridge UK; ^2^ Department of Biostatistics Virginia Commonwealth University Richmond Virginia USA

**Keywords:** combination trials, dose‐finding, ordering specification, partial ordering continual reassessment methods

## Abstract

Recent years have seen increasing popularities in therapies that combines several drugs and/or schedules, which imposes great difficulties on the design of appropriate Phase I clinical trials. The Partial Ordering Continual Reassessment Method (POCRM), among others, is a popular design that can be easily adapted to those complex settings. However, the design is introduced and evaluated in the setting of the combination of two drugs, and the combination of more than two drugs has rarely been investigated. In particular, the specification of toxicity orderings has always been an essential part of the POCRM, which becomes more difficult in high‐dimensional settings due to the combinatorially increasing nature of the number of possible orderings of combination/schedules. This article proposes a systematic approach to specify orderings based on asymptotic properties in the setting of the combination of more than two drugs. Large simulation studies show that this novel ordering specification method leads to better design performance both asymptotically and with finite sample sizes.

## 1 Introduction

Drug combination therapy, which involves different drugs targeting different therapeutic pathways, aims to improve treatment response, reduce resistance development, and minimize adverse events. It has emerged as one of the most important precision treatment options against cancer. In a review of 465 National Cancer Institute‐sponsored phase I trials for solid tumors conducted between 2000 and 2019, Chihara et al. [1] reported that 69% of them used combination therapies. These combination therapies yielded substantially higher response rates than monotherapy (15.8% vs. 3.5%), while maintaining similar rates of adverse events.

Despite the improved treatment efficacy with combination drugs identifying the maximum tolerated dose combination (MTC), defined as the dose combination with a dose‐limiting toxicity (DLT) probability closest to a pre‐specified target toxicity level (TTL), remains a challenge in early‐phase drug combination trials. Key difficulties include small sample sizes, large and complex combination spaces, and unknown drug–drug interactions. Unlike single‐agent trials, where toxicity typically increases with dose levels, and thus the ordering between the DLT probabilities of doses is fully known, combination trials often involve unknown orderings among some of the dose‐combinations being studied. For example, comparing a combination with a higher dose of drug A to a combination with a higher dose of drug B does not yield an obvious ordering among their DLT probabilities. More explicitly, the term “ordering” refers to a relationship of the DLT risks between any two combinations being studied. As the number of drugs or dose levels increases, the number of possible orderings increases sharply [2].

Numerous statistical designs have been proposed for dual‐agent trials. Reference [3] proposed a method to identify the MTCs by fitting a six‐parameter model. Reference [4] developed an isotonic design based on simple and partial orderings for two‐drug combination trials by classifying the nodal and non‐nodal parameters. Reference [5, 6] proposed a Bayesian adaptive design based on a copula‐type model to estimate the DLT probabilities of drug combinations, as well as another design based on latent contingency tables. Reference [7] extended the one‐dimensional Bayesian logistic regression model to the two‐dimensional case with an interaction multiplier to model the drug‐drug interactions. Reference [8] extended the Bayesian optimal interval (BOIN) design for combination trials, in which the posterior probability that the DLT probability of the selected dose pair falls into a pre‐specified interval is maximized. Reference [9] extended the Keyboard design to handle drug combination trials. Reference [10] proposed a model‐free design by parametrizing the ratios between each neighboring combination using independent Beta distributions and assuming the ratios are independent. Reference [11] proposed a curve‐free design for dual‐agent trials using the product of independent Beta probabilities escalation (PIPE) strategy to identify the MTC contour.

However, these approaches are primarily tailored to dual‐agent trials and might not be straightforward to generalize to more complex combination structures. On the other hand, [12] developed the partial order continual reassessment method (POCRM), which generalizes well to not only the combination of more than two agents, but any settings with uncertainties in the ordering between the treatment regimens, such as the combination of dose with different schedules [13]. In the nutshell, the POCRM starts by listing a small number of possible orderings of the dose‐combinations being studied, reflecting potential feasible orderings between combinations. Then, the Bayesian model selection technique is adopted to select the ordering most compatible with the observed data. Under the selected ordering, the continual reassessment method [CRM, O'Quigley et al. [14]] can then be applied to selected the MTC.

While existing dual‐agent designs can, in theory, be extended to accommodate triple combinations, there is currently no studies evaluating their performance in these more complex settings. As the number of study drugs increases, the total number of dose combinations and potential DLT probability orderings grows rapidly. However, the sample size in phase I trials typically remains small, which further complicates the identification of the MTC. For instance, in a dual‐agent setting where agents A and B each have 2 dose levels, there are 4 combinations with 2 possible orderings. Introducing a third agent with 3 dose levels increases the total to 12 combinations with approximately 2000 possible orderings.

Saha et al. [15] also studied the orderings for the PCORM, where they pointed out that the monotonicity assumption is not necessary for the CRM to be consistent. They then use this result to explain certain behaviors of the POCRM. Explicitly, since the monotonicity is not necessary, the POCRM could perform well under an incorrect ordering if their relaxed condition is satisfied. However, although providing some useful insights to the POCRM, their explanation depends on knowing the true toxicity probabilities and the location of the MTC. While this is insightful and does help with better understanding of the POCRM behavior, in this manuscript, we take a step further and provide practical recommendations on the ordering specification for the POCRM, which has not been done before.

The ordering specification recommended in this article is based on asymptotic consistency, that is, the probability of recommending the correct MTC should converge to 1 as the sample size goes to infinity. The method is originated in Chen and Mozgunov [16] in the context of a two‐dimensional drug‐drug combination problem. The original method requires explicitly listing out all possible orderings, which becomes impossible for most of the three‐dimensional problems. For example, when the three agents each have 2, 3, and 4 levels, it takes around 45 min for a regular laptop to list the first around 2 million orderings before it runs out of memory space. Hence, it becomes crucial to have a computationally feasible and efficient method to specify orderings. The Adding‐Refining algorithm recommended in this article recommends a small number of consistent orderings without requiring the full list. Having a small number makes it easy to communicate to the clinicians. In order for the design to be implemented in real trials, the clinicians would need to go through all the orderings included and understand what these orderings mean, as well as what the differences are between any two orderings. Moreover, the computation is much cheaper with less orderings, which makes it feasible for statisticians to explore the design performance under various design parameters and scenarios of combination‐toxicity relationships.

The work proposed in this article is motivated by two trials, one that combines three drugs involving 12 dose triplets and the other that combines two drugs with multiple treatment schedules for a total of 32 dose‐schedule triplets. We adopt the POCRM framework because of its flexibility and adaptability to higher‐dimensional dose spaces. However, one essential step of the POCRM is to specify a small set of possible orderings. Listing all possible orderings is only feasible when the number of study drugs and dose levels is relatively small. For example, two drugs with up to 3 levels of each drug. Reference [17] established a set of sufficient conditions under which the POCRM is asymptotically consistent in the two‐dimensional setting, that is, the probability of selecting the correct MTC converges to 1 as the sample size goes to infinity. Here, we extend their approach to ordering specification to the three‐dimensional case and apply POCRM for the selected orderings to the motivating application. Our work is the first to identify the MTC for triple combinations, and our simulation studies show that the percentage of correct selection (PCS) of the MTC converges to 1 as the sample size increases. We have also studied the performance of the POCRM using the proposed ordering specification techniques under finite sample sizes in triplet combination studies. Our approach can be easily applied to handle more complex cases involving more study drugs without developing new methods.

## 2 Motivating Trials

This article is motivated by two real trials. The first one is a proposed study from the Stanford Cancer Institute investigating a triple combination therapy in pediatric cancer, with a target toxicity rate of 0.25 to define the MTC. Although the study team ultimately revised the trial design to accommodate a more limited scope, the original design—tailored to the full set of planned combinations—is described here. The initial proposal aimed to evaluate the safety of three investigational agents—Drug A, Drug B, and Drug C. Drug A has three dose levels (400, 600, and 1000 mg), Drug B has four dose levels (10, 20, 30, and 40 mg), and Drug C has two dose levels (600 and 1200 mg). We are interested in 12 possible combinations induced by these drugs (see Table 1). The trial starts with the combination in which Drugs A, B, and C are administered at 400 mg, 10 mg, and 600 mg, respectively. The dose‐escalation process follows a two‐stage approach [18]. In the first stage, a rule‐based escalation strategy is employed: small cohorts of size 1 are sequentially assigned to drug combinations based on a pre‐specified path. This stage continues until the first DLT is observed, at which point the design transitions to a model‐based dose allocation approach for the remainder of the trial. This motivating example illustrates the complexity of dose‐escalation trials involving more than two agents, where the combinatorial nature of dosing leads to a high‐dimensional space with numerous partially known toxicity relationships.

**TABLE 1:** Toxicity array for the combination of 3 drugs.

				Drug A		
				400	600	1000
Drug C	1200	Drug B	40		${\tilde{d}}_{2,4,2}$	${\tilde{d}}_{3,4,2}$
			30		${\tilde{d}}_{2,3,2}$	${\tilde{d}}_{3,3,2}$
			20		${\tilde{d}}_{2,2,2}$	
			10			
	600	Drug B	40		${\tilde{d}}_{2,4,1}$	${\tilde{d}}_{3,4,1}$
			30		${\tilde{d}}_{2,3,1}$	${\tilde{d}}_{3,3,1}$
			20	${\tilde{d}}_{1,2,1}$	${\tilde{d}}_{2,2,1}$	
			10	${\tilde{d}}_{1,1,1}$		

The other motivating trial considers the combination of two new experimental drugs in oncology that has never been used before with various schedules of administration. Two potential levels of the first drug (2.5, 5 mg) is combined with four potential levels of the second drug (15, 30, 45, 75 mg). Then, these 8 combinations are further combined with four different schedules, where the toxicity relationship between the schedules is known and is monotonically increasing. These gives 32 treatment regimens and over a billion possible orderings to choose from.

In general, the three‐dimensional problem rises whenever the administration schedule has been taken into account in a drug‐combination trial. Mozgunov et al. [19] provides more such real‐world examples.

## 3 Methodology

This section starts by briefly introducing the POCRM design under the setting of the combination of three treatments in Section 3.1. Section 3.2 gives a set of sufficient condition which ensures the asymptotic consistency of the POCRM. Then, Section 3.3 and 3.4 suggest methods to specify orderings based on consistency.

### 3.1 The Partial Ordering Continual Reassessment Method (POCRM)

This section describes the two‐stage likelihood‐based POCRM design [18] under the setting of the first motivating trial introduced in Section 2. The three drugs are denoted as drug A with dose levels $a_{1}<a_{2}<a_{3}$, drug B with levels $b_{1}<b_{2}<b_{3}<b_{4}$, and drug C with levels $c_{1}<c_{2}$. The 12 treatment combinations are arranged into a toxicity array shown in Table 1, where each combination is denoted as ${\tilde{d}}_{i,j,k}=(a_{i},b_{j},c_{k})$ for i=1,2,3, j=1,…,4, k=1,2. The array is divided into the lower and upper part by the level of drug C. Within each part, moving from the bottom‐left to the top‐right would increase the toxicity. For example, the sequence ${\tilde{d}}_{1,1,1}\to {\tilde{d}}_{1,2,1}\to {\tilde{d}}_{2,2,1}\to {\tilde{d}}_{2,3,1}$ has increasing toxicities. Then, fixing the level of A and B, the combination in the upper part has toxicity higher than the one in the lower part. For example, ${\tilde{d}}_{2,2,2}$ is more toxic than ${\tilde{d}}_{2,2,1}$.

The monotonicity of each single drug implies *partial ordering* of the drug‐combinations, where “partial” refers to the subsets of the combination space whose toxicity ordering is known. Any ordering of the whole the combination space respecting the known partial ordering is called a possible “complete” ordering. As an example, in Table 1, the toxicity ordering between ${\tilde{d}}_{2,3,1}$ and ${\tilde{d}}_{3,3,1}$ is known because they have the same level of drug B and C, and ${\tilde{d}}_{3,3,1}$ has a higher level of drug A than ${\tilde{d}}_{2,3,1}$. However, the ordering between ${\tilde{d}}_{2,4,1}$ and ${\tilde{d}}_{3,3,1}$ is unknown because ${\tilde{d}}_{2,4,1}$ has a higher level of drug B but lower level of drug A then ${\tilde{d}}_{3,3,1}$. The possible complete orderings are denoted as ${𝒪}_{1},\ldots ,{𝒪}_{M^{*}}$, where $M^{*}$ is the total number of possible orderings.

The POCRM enrolls patients sequentially and has two stages as follows.

• Stage 1: patients are enrolled to a pre‐specified sequence of combinations until the first toxicity event has been observed.
• Stage 2:
  - Ordering selection: fit the CRM under each ordering ${𝒪}_{m}$, $m=1,\ldots ,M^{*}$, calculate the posterior probability of ${𝒪}_{m}$, p(m|Data)∝p(m)ℙ(Data|m), where p(m)≥0, $\sum_{m}p(m)=1$, is the prior probability of ordering m, and ℙ(Data|m) denotes the marginal likelihood under ordering m. The choice of p(m) will be discussed in Section 6. The ordering ${𝒪}_{m^{*}}$ with $p(m^{*}|\text{Data})=\max_{m=1,\ldots ,M^{*}}p(m|\text{Data})$ is selected.
  - Combination selection: apply the CRM under ordering ${𝒪}_{m^{*}}$ to estimate the toxicity probability of each combination ${\hat{p}}_{i,j,k}$. Enroll the next cohort of patients to ${\tilde{d}}_{i^{*},j^{*},k^{*}}$ with ${\hat{p}}_{i^{*},j^{*},k^{*}}$ closest to the TTL.

However, the number of possible complete orderings, $M^{*}$, increases combinatorially with the number of dose levels of each drug. Even under only 2 drug each with 4 levels, that is, a 4×4 grid, $M^{*}=24024$. For the combination of 3 drugs, $M^{*}$ increases more rapidly. When each drug has only 2 level, that is, 2×2×2 grid, $M^{*}=48$, which increases to $M^{*}=2163$ under the 2×2×3 grid and $M^{*}=183002$ under the 2×2×4 grid. Hence, it is infeasible to include all possible orderings into the POCRM, and a method to specify which orderings to include is essential. In general, the orderings can either be specified based on clinical information [19] or based on statistical considerations. The latter looks for the set of orderings that gives the best operational characteristics (OC) under a wide range of possible scenarios of toxicity probabilities. Reference [20] suggests 6 orderings for the combination of 2 drugs based on statistical considerations, referred to as the *Wages 6 orderings*, which has then become the convention for ordering specification of the POCRM.

Nevertheless, it has been shown that the Wages 6 orderings can results in particularly low accuracy when the MTC is in the middle of the grid under the combination of two drugs, that is, both drugs take the middle level of their possible levels [16]. Furthermore, there is no direct way to translate these 6 orderings to the combination of more than 2 agents. Hence, an alternative suggestion of specifying orderings based on limiting behaviors (i.e., asymptotic consistency) has been suggested [16]. This is introduced in Section 3.2 below.

### 3.2 The Consistency of the POCRM

The notion of consistency considers the design behavior as the sample size goes to infinity.

Definition 1 (Asymptotic consistency) A dose‐finding design is asymptotically consistent if the probability of selecting the MTC converges to 1, almost surely, as the sample size N goes to infinity.

Reference [16] gave a set of sufficient conditions for the consistency of the POCRM, which includes guidance on the ordering specification. We first introduce the technique of *relabeling* the combinations.

Definition 2 (Relabeling of combinations) Define the relabeling operator L, which maps the combination ${\tilde{d}}_{i,j,k}$ to its label $d_{l}$ given the scenario of true toxicity probabilities R, i=1,…,I; j=1,…,J; k=1,…,K; l=1,…,I·J·K. Upon relabeling, the toxicity probabilities increase in the order $d_{1}\to d_{2}\to \ldots \to d_{L}$, where $d_{1}$ is the least toxic combination, and $d_{L}$ is the most toxic combination.

Under a given scenario of toxicity probabilities R, a necessary condition for the POCRM to be consistent is that at least one ordering from the *correct ordering group* of R has been included, which is defined as follows.

Definition 3 (correct ordering group) Under a given scenario R with a single MTC ${\tilde{d}}_{i^{*},j^{*},k^{*}}$, let $d_{\nu }=L({\tilde{d}}_{i^{*},j^{*},k^{*}};R)$ be the label of the MTC upon relabeling. Let 𝒪[l] be the lth toxic combination under ordering 𝒪, and 𝒪[1:l]={𝒪[1],𝒪[2],…,𝒪[l]}. Then, ordering 𝒪 belongs to the correct ordering group of R if and only if

1. $𝒪[\nu ]=d_{\nu }={\tilde{d}}_{i^{*},j^{*},k^{*}}$, that is, the MTC is ordered correctly;
2. $𝒪[1:(\nu -1)]=\{d_{1},\ldots ,d_{\nu -1}\}$, that is, all combinations less toxic than the MTC are ordered before the MTC.

In words, an ordering belongs to the correct group if the MTC is at its correct location, all combinations less toxic than the MTC are ordered before the MTC (regardless of the ordering amongst themselves), and all combinations more toxic than the MTC are ordered after the MTC (regardless of the ordering amongst themselves). Intuitively, if a more toxic combination is ordered before the MTC, then the POCRM will have difficulty escalating past this combination. Conversely, if a less toxic combination is ordered after the MTC, once the design goes to this combination, it will have difficulty de‐escalating back to the MTC.

Theorem 1 *Given the true toxicity scenario* R *and the toxicity skeleton* α *that satisfies the POCRM consistency condition in Chen and Mozgunov* [16], *a necessary and sufficient condition for the consistency of the POCRM is that at least one ordering in the correct ordering group of* R *has been included into the POCRM*.

Intuitively, if there is no ordering from the correct ordering group, the POCRM will either be stuck at an overly toxic combination before the MTC or it will stay at a less toxic combination and cannot move back to the MTC. Conversely, given at least one ordering from the correct group, a well‐selected toxicity skeleton can lead to consistency of the POCRM.

The above definitions are illustrated in Section 4.1, and a graphical illustration in the simple 2×2×2 grid has been provided in the Supporting Information.

### 3.3 Ordering Specification by Listing All Orderings

In practice, the true toxicity scenario is unknown and thus Theorem 1 cannot be directly applied. The key to solve this problem is to realize that the correct ordering group is not characterized by the toxicity scenarios, but *order‐scenarios* defined as follows.

Definition 4 (Order‐scenarios) Let $R^{\left(1\right)},R^{\left(2\right)}$ be two toxicity scenarios with the same MTC ${\tilde{d}}_{i^{*},j^{*},k^{*}}$. Let $d_{\nu_{1}}=L({\tilde{d}}_{i^{*},j^{*},k^{*}};R^{\left(1\right)})$ and $d_{\nu_{2}}=L({\tilde{d}}_{i^{*},j^{*},k^{*}};R^{\left(2\right)})$ be the labels of the MTC under the two toxicity scenarios. Define the set of combinations with toxicity probabilities below the MTC $B_{c}=\{{\tilde{d}}_{i,j,k}:L({\tilde{d}}_{i,j,k};R^{\left(c\right)})=d_{l},l<\nu_{c}\}$, c=1,2, referred to as the “below MTC set”. Then, $R^{\left(1\right)}$ and $R^{\left(2\right)}$ belong to the same order‐scenario if

$$1.\;\nu_{1}=\nu_{2};\;\;2.\;B_{1}\equiv B_{2}.$$

In words, there are three elements that define an order‐scenario, (1) the MTC, (2) the label of the MTC, and (3) the combinations with toxicity below the MTC. Elements (1) and (3) together also determines the combinations more toxic than the MTC. Hence, these are exactly the elements that define a correct ordering group. This is the information conveyed by Proposition 1.

Proposition 1 (Correct ordering groups are characterized by order‐scenarios) *Given two toxicity scenarios* $R^{\left(1\right)}$ *and* $R^{\left(2\right)}$ *belonging to the same order‐scenario. If ordering* 𝒪 *belongs to the correct ordering group under* $R^{\left(1\right)}$, *then it also belongs to the correct ordering group under* $R^{\left(2\right)}$.

By construction, if $R^{\left(1\right)}$ and $R^{\left(2\right)}$ belongs to the same order‐scenario, they have a common MTC ${\tilde{d}}_{i^{*},j^{*},k^{*}}$ with common label $d_{\nu }$, and the below MTC sets $B_{1}=B_{2}$.

If 𝒪 belongs to the correct group of $R^{\left(1\right)}$,

1. $𝒪[\nu ]={\tilde{d}}_{i^{*},j^{*},k^{*}}$,
2. $𝒪[1:(\nu -1)]=B_{1}=B_{2}$.

Hence, 𝒪 belongs to the correct group under $R^{\left(2\right)}$.

Hence, if the true toxicity scenario is unknown, it suffices to consider all possible order‐scenarios, and ensure at least one ordering in each correct group has been included, as formalized in Proposition 2. The advantage of considering order‐scenarios instead of the toxicity scenarios is that there are only finitely many of them, as opposed to the infinitely many toxicity scenarios. Hence, it becomes feasible to list out all of the order‐scenarios, and check Theorem 1 under each possible order‐scenario.

Proposition 2 (Ordering specification with unknown toxicity scenario) *When the true toxicity scenario* R *is unknown, consider all possible order‐scenarios* ${\tilde{R}}^{\left(1\right)},\ldots ,{\tilde{R}}^{\left(c\right)}$. *If there exist at least one ordering in the correct ordering group of* ${\tilde{R}}^{\left(c\right)}$, ∀c=1,…,C, *then the chosen set of orderings is consistent*.

### 3.4 Ordering Specification Without Listing All Orderings

One practical concern to apply Proposition 2 is that the number of possible orderings, $M^{*}$, can be large, which makes it computationally infeasible to list out all of them. In particular, $M^{*}$ increases combinatorially with the number of dose levels of each drug. Hence, here we propose an approach to specify a set of consistent orderings without having to list all $M^{*}$ possible orderings.

To start with, given a scenario R, an ordering belongs to the correct ordering group under R can be constructed in the way suggested by Lemma 1.

Lemma 1 (Construct correct orderings) *Given any scenario* R, *let* ${\tilde{d}}_{i^{*},j^{*},k^{*}}$ *be its MTC*, $d_{\nu }=L({\tilde{d}}_{i^{*},j^{*},k^{*}};R)$, *and* $B=\{{\tilde{d}}_{i,j,k}:L(d_{i,j,k};R)<\nu \}$. *Then, construct ordering* 𝒪 *in the following steps*.

1. *Fix* $𝒪[\nu ]={\tilde{d}}_{i^{*},j^{*},k^{*}}$.
2. *Take a random ordering of* B *respecting the known partial orderings, and put into* 𝒪[1:(ν−1)].
3. *Take a random ordering of the remaining combinations respecting the known partial orderings, and put into* 𝒪[(ν+1):L].

*Then, ordering* 𝒪 *belongs to the correct ordering group under scenario* R.

By construction, $𝒪[\nu ]={\tilde{d}}_{i^{*},j^{*},k^{*}}$ and 𝒪[1:(ν−1)]=B.

Since the number of order‐scenarios is much smaller than the number of possible orderings, the orderings could be constructed based on the full list of order‐scenarios. Hence, we propose the *Adding‐Refining* algorithm, shown in Algorithm 1. The *Adding* step starts with any possible ordering ${𝒪}_{1}$, and progressively checks Theorem 1 under each order‐scenario. Given the MTC ${\tilde{d}}_{i,j,k}$, the order‐scenarios ${\tilde{R}}_{i,j,k}$ are found by simulating all possible below MTC sets B. Then, under each order‐scenario, count the number of orderings in its correct group among the current list of orderings, n. If n=0, this order‐scenario has not been covered, and an ordering from the correct group, constructed by Lemma 1, is added to list. Then, the *Refining* step aims to refine the list of orderings obtained by the *Adding* step by taking subsets of S orderings and checking Proposition 2. The smallest subset will be retained as the final choice of orderings.

ALGORITHM 1 **The Adding‐Refining algorithm for ordering specification.**

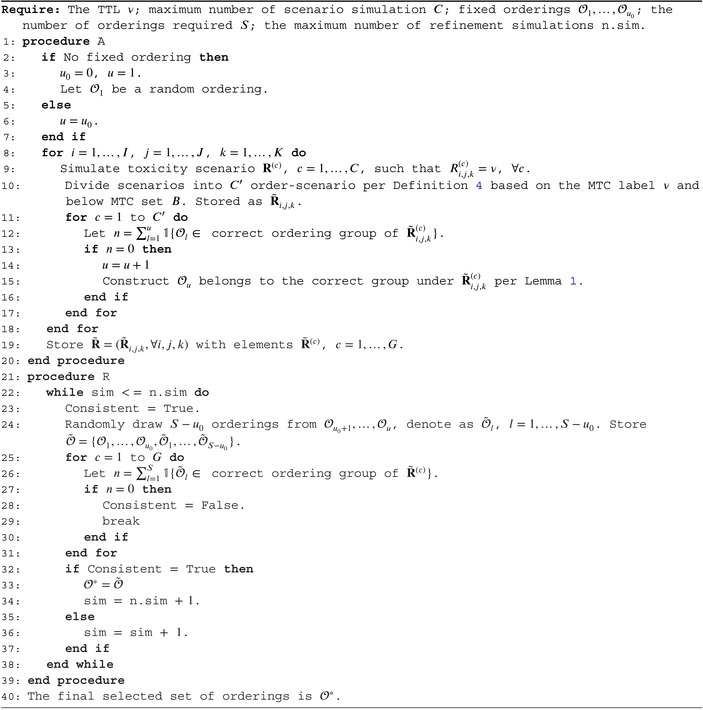

## 4 Application of the Ordering Specification Method to the Motivating Trial

This section applies the consistency conditions and ordering specification approach introduced in Section 3 to the motivating trial introduced in Section 2. In this case, there are 148 possible orderings, listed in the Supporting Information [21]. Although the full list is available, the Adding‐Refining algorithm will also be applied, and the outputs from the algorithm will be compared with the list obtained from the full list.

### 4.1 Correct Ordering Group

This section gives an example of the correct ordering group per Definition 3 using scenario $R^{\left(2\right)}$ in Table 3. The TTL is θ=0.25, and the MTC is ${\tilde{d}}_{2,2,2}$ (highlighted in **bold**). After relabeling per Definition 2, the labels are

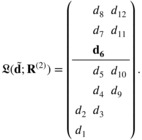

Consider two orderings, ordering 27 and 50 (the full list of 148 orderings are given in the Supporting Information),

$$\begin{array}{ll} {𝒪}_{27} & :{\tilde{d}}_{1,1,1}\to {\tilde{d}}_{1,2,1}\to {\tilde{d}}_{2,2,1}\to {\tilde{d}}_{2,3,1}\to {\tilde{d}}_{2,4,1}\to {\tilde{d}}_{3,3,1}\to {\tilde{d}}_{3,4,1}\\ & \;\to {\tilde{d}}_{2,2,2}\to {\tilde{d}}_{2,3,2}\to {\tilde{d}}_{3,3,2}\to {\tilde{d}}_{2,4,2}\to {\tilde{d}}_{3,4,2},\\ {𝒪}_{50} & :{\tilde{d}}_{1,1,1}\to {\tilde{d}}_{1,2,1}\to {\tilde{d}}_{2,2,1}\to {\tilde{d}}_{2,3,1}\to {\tilde{d}}_{2,4,1}\to {\tilde{d}}_{2,2,2}\to {\tilde{d}}_{2,3,2}\\ & \;\to {\tilde{d}}_{3,3,1}\to {\tilde{d}}_{2,4,2}\to {\tilde{d}}_{3,3,2}\to {\tilde{d}}_{3,4,1}\to {\tilde{d}}_{3,4,2}, \end{array}$$

or alternatively, the orderings can be expressed in the form

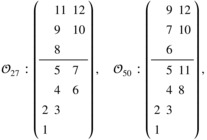

where the numbers in the array correspond to the position of the combination in the ordering. For example, in the LHS array, the number 5 means ${𝒪}_{27}[5]={\tilde{d}}_{2,4,1}$, that is, ${\tilde{d}}_{2,4,1}$ is the 5th toxic combination under ordering ${𝒪}_{27}$.

Under scenario $R^{\left(2\right)}$, the MTC gets label $L({\tilde{d}}_{2,2,2};R^{\left(1\right)})=d_{6}$, (ν=6 per Definition 3), and the two orderings have ${𝒪}_{27}[6]={\tilde{d}}_{3,3,1}=d_{9}$, ${𝒪}_{50}[6]={\tilde{d}}_{2,2,2}=d_{6}$. Hence, the first condition in Definition 3 is satisfied by ${𝒪}_{50}$ but not ${𝒪}_{27}$. ${𝒪}_{50}[1:(\nu -1)]={𝒪}_{50}[1:5]=\{d_{1},d_{2},d_{3},d_{4},d_{5}\}$, and thus ordering 50 also satisfies the second condition in Definition 3. Hence, overall, only ordering ${𝒪}_{50}$ belongs to the correct group under scenario $R^{\left(2\right)}$.

**FIGURE 1:**
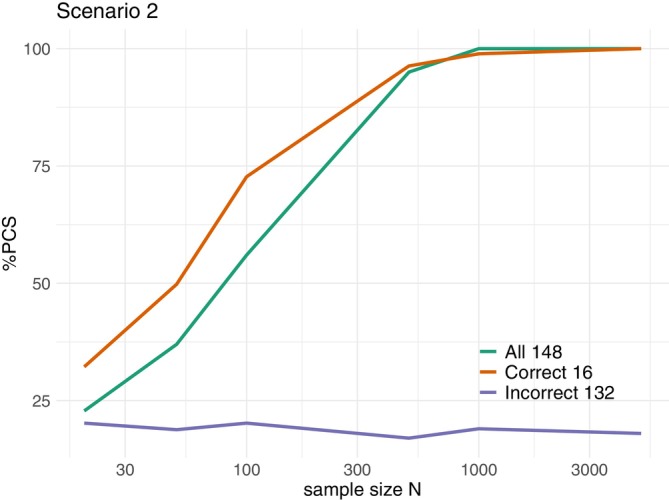
POCRM under all 148 orderings (green), 16 correct orderings (orange), 132 incorrect orderings (purple). All estimations based on $10^{4}$ simulations.

Among all 148 possible complete orderings, ordering 37‐52 belong to the correct group under scenario $R^{\left(2\right)}$. Figure 1 below plots the PCS of the POCRM against sample sizes under 3 choices of ordering: (1) all 148 orderings; (2) 16 correct orderings, ordering 37‐52; (3) 132 incorrect orderings, all but ordering 37‐52. It shows that the POCRM under all 148 orderings is consistent, with PCS converging to 100% at sample size 1000. If only the 16 orderings in the correct group are included, the POCRM is also consistent, and the PCS at small sample sizes are higher than with 148 orderings by around 10%. However, if none of the orderings in the correct group is included, the PCS remains around 20% no matter how many patients are enrolled.

### 4.2 Scenario‐Agnostic Orderings From the Full List

**FIGURE 2:**
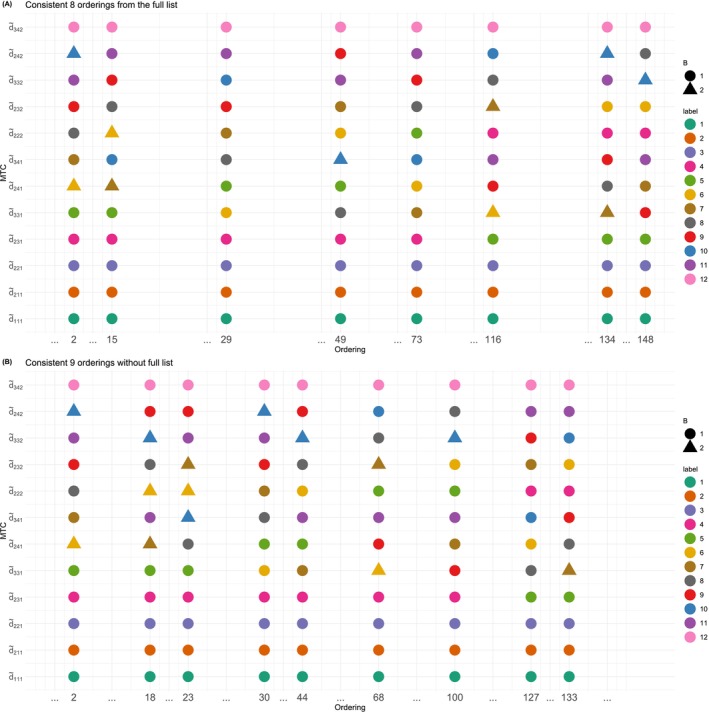
Order‐scenarios.

The term “scenario‐agnostic” refers to the case when the true toxicity scenarios are unknown. We first find the smallest number of orderings satisfying Proposition 2 by listing out all 148 orderings. In total, there are 47 order‐scenarios, which are graphically shown in Figure 2. The three elements that define an order‐scenario are the MTC, shown by the y‐axis, the label of the MTC, shown by colors of the dots, and the below MTC set B, shown by the shapes of the dots. Combinations of these three defines a unique order‐scenario, and the corresponding x‐axis are the orderings belonging to the correct group under this order‐scenario. For example, the order‐scenario shown by the green circle on the bottom row has MTC ${\tilde{d}}_{1,1,1}$, which is labeled $d_{1}$ and there is only 1 possible below MTC set B, which is the empty set. When the MTC is ${\tilde{d}}_{3,3,1}$ and labeled as $d_{6}$, there are two possible below MTC sets, $\left\{{\tilde{d}}_{1,1,1},{\tilde{d}}_{1,2,1},{\tilde{d}}_{2,2,1},{\tilde{d}}_{2,3,1},{\tilde{d}}_{2,4,1}\right\}$ or $\left\{{\tilde{d}}_{1,1,1},{\tilde{d}}_{1,2,1},{\tilde{d}}_{2,2,1},{\tilde{d}}_{2,3,1},{\tilde{d}}_{2,2,2}\right\}$. These defined the two order‐scenarios, shown by the yellow circle and triangle on the 6th row from the bottom. The selected orderings, shown by columns, should cover all 47 possible order‐scenarios (types of dots).

To select the smallest number of orderings satisfying the consistency condition, the R function SpecifyOrder() is used, which will be illustrated in details in Section 4.4. The function repeatedly takes subsets of S∈ℕ orderings and check whether the consistency conditions are satisfied. Starting with S=1, if a consistency subset cannot be obtained after $10^{5}$ attempts, increase S by one and repeat the procedure. In our example, the 8 orderings, ordering 2, 15, 29, 49, 73, 116, 134, 148, shown in (Panel A) is the smallest number of orderings that satisfies the consistency condition.

### 4.3 Scenario‐Agnostic Orderings Without Listing All Orderings

Without having to list out all 148 possible orderings, the Adding‐Refining algorithm, Algorithm 1, has been applied to obtain consistent set of orderings. The maximum number of simulated scenarios is set to C=10,000.

In the Adding step, the algorithm started with $u_{0}=0$ and u=1, that is, no fixed ordering, and ${𝒪}_{1}={\tilde{d}}_{1,1,1}\to {\tilde{d}}_{1,2,1}\to {\tilde{d}}_{2,2,1}\to {\tilde{d}}_{2,3,1}\to {\tilde{d}}_{3,3,1}\to {\tilde{d}}_{2,4,1}\to {\tilde{d}}_{3,4,1}\to {\tilde{d}}_{2,2,2}\to {\tilde{d}}_{2,3,2}\to {\tilde{d}}_{3,3,2}\to {\tilde{d}}_{2,4,2}\to {\tilde{d}}_{3,4,2}$. The 47 order‐scenarios will be considered sequentially. Under each order‐scenario, the algorithm check whether its correct ordering group has been covered. If not, a random ordering from this group will be added to the list of selected orderings. For example, if the algorithm starts with the order‐scenario shown by the green circle at the bottom row in Figure 2, the correct group contains all 148 orderings, which has been covered by ${𝒪}_{1}$. Hence, u is unchanged, and the algorithm moves to the next order‐scenario, the light green dot on the 4th row from the bottom, for instance. This corresponds to ${\tilde{d}}_{2,3,1}$ being the MTC, labeled as $d_{5}$, and the below MTC set $B=\{{\tilde{d}}_{1,1,1},{\tilde{d}}_{1,2,1},{\tilde{d}}_{2,2,1},{\tilde{d}}_{2,2,2}\}$. The current ${𝒪}_{1}$ does not belong to its correct group because ${𝒪}_{1}[\nu ]={𝒪}_{1}[5]={\tilde{d}}_{3,3,1}$, not the MTC. Hence, a random ordering from the correct group is added, for example, ${𝒪}_{2}={\tilde{d}}_{1,1,1}\to {\tilde{d}}_{1,2,1}\to {\tilde{d}}_{2,2,1}\to {\tilde{d}}_{2,2,2}\to {\tilde{d}}_{2,3,1}\to {\tilde{d}}_{3,3,1}\to {\tilde{d}}_{2,3,2}\to {\tilde{d}}_{3,3,2}\to {\tilde{d}}_{2,4,1}\to {\tilde{d}}_{2,4,2}\to {\tilde{d}}_{3,4,1}\to {\tilde{d}}_{3,4,2}$, and u=2.

After all the 47 order‐scenarios are considered, the algorithm accumulated 28 orderings, which are subsequently entered into the Refining step. The smallest subset respecting the consistency condition has 9 orderings, which are orderings 2, 18, 23, 30, 44, 68, 100, 127, and 133. These are shown in (Panel B) in Figure 2, it can be checked that all 47 types of dots have been covered at least once.

Under the example grid with 148 possible orderings and 47 order‐scenarios, the ratio is only around 3, which may make the Adding‐Refining algorithm unnecessary. Table 2 below summarizes the number of possible orderings, order‐scenarios, and orderings needed to ensure consistency under various grids. The ratios calculated are the ratio between the number of all possible orderings to the number of order‐scenarios (4th row) and the number of orderings needed (last row). It can be seen that the ratio between the number of orderings and the number of order‐scenarios grows combinatorially. Increasing the level of any drug by one level leads to increasing the ratio by more than 10 folds. In particular, the 2×3×4 grid gives an example where it is not computationally feasible to list all orderings. It takes around 45 min for a regular laptop to list the first around 2 million orderings before it runs out of memory space, but listing all 1470 order‐scenarios takes only around 5 min.

**TABLE 2:** Number of orderings, order‐scenarios, orderings needed to ensure consistency under various grids.

	3‐dimensional problems				2‐dimensional problems			
Grid	2×2×2	2×2×3	2×3×4		2×2	3×3	3×4	4×4
# orderings	48	2452	>2 million		2	42	462	24024
# order‐scenarios (ratio)	32	100	1470		2	30	34	88
	1.5	24.5	>1500		1	1.5	13.6	273
# orderings needed (ratio)	8	30	232		2	6	15	43
	6	81.7	>8600		1	7	30.8	558.7

### 4.4 Practical Implementation

The R function Adding() is provided to perform the Adding step of the Adding‐Refining algorithm, which also lists out all possible order‐scenarios. The function takes the following arguments.

- dim: the dimension of the dose‐combination grid.
- C: the number of scenario simulations, as defined in the Adding‐Refining algorithm.
- MTC: the list of MTCs.

The function outputs a set of ordering that will be subsequently entered into the Refining step. Moreover, it provides all possible order‐scenarios.

An R function SpecifyOrder() is provided for ordering specification, which takes the following set of arguments.

- scen: Q×L matrix, each row contains one scenario.
- orderings: the set of orderings to choose from.
- M: the number of orderings to include;
- order.fix: optional, a small number of orderings users wish to fix;

The optional argument order.fix comes from discussions with clinicians. Clinical knowledge of the drug‐combinations may lead to certain orderings being more likely than the others, in which case, the function can ensure these orderings will be included. For example, clinicians may determine that drug A is much more toxic than drug B, and drug B is more toxic than drug C. This clinical belief translates to the ordering ${\tilde{d}}_{1,1,1}\to {\tilde{d}}_{1,2,1}\to {\tilde{d}}_{2,2,1}\to {\tilde{d}}_{2,2,2}\to {\tilde{d}}_{2,3,1}\to {\tilde{d}}_{2,3,2}\to {\tilde{d}}_{2,4,1}\to {\tilde{d}}_{2,4,2}\to {\tilde{d}}_{3,3,1}\to {\tilde{d}}_{3,3,2}\to {\tilde{d}}_{3,4,1}\to {\tilde{d}}_{3,4,2}$, which should be put into order.fix to ensure being included in the output of M orderings.

For scenario‐specific ordering specifications, scen is the set of scenarios of interest. For scenario‐agnostic ordering specifications, scen contains all possible order‐scenarios. If the full list of $M^{*}$ possible complete orderings is available, orderings is this full list. If the Adding‐Refining algorithm is used, orderings is the output from the Adding step. Finally, note that if M is too small, there is no guarantee that consistency can be achieved with M orderings. All codes can be found on GitHub https://github.com/WeishiC/POCRM‐with‐Triple‐Combinations.

## 5 Simulations

### 5.1 Setting

Simulation studies are provided to evaluate the ordering specification methods proposed in Section 3, the setting in the motivational trial introduced in Section 2 is used, where three drugs and 12 possible combinations are investigated. The likelihood‐based POCRM will be used, patients are enrolled sequentially, and the escalation path in stage one will be ${\tilde{d}}_{1,1,1}\to {\tilde{d}}_{1,2,1}\to {\tilde{d}}_{2,2,1}\to {\tilde{d}}_{2,3,1}\to {\tilde{d}}_{3,3,1}\to {\tilde{d}}_{2,4,1}\to {\tilde{d}}_{3,4,1}\to {\tilde{d}}_{2,2,2}\to {\tilde{d}}_{2,3,2}\to {\tilde{d}}_{3,3,2}\to {\tilde{d}}_{2,4,2}\to {\tilde{d}}_{3,4,2}$. The target toxicity level (TTL) is set to 0.25. The toxicity skeleton used is α=(0.0003, 0.02, 0.04, 0.08, 0.19, 0.25, 0.28, 0.31, 0.38, 0.44, 0.50, 0.56). The skeleton is calibrated based on the conditions in [16] so that the POCRM design under all 148 orderings is consistent.

### 5.2 Scenarios

The 12 scenarios in Table 3 are used to evaluate the performance of the proposed ordering specifications. Each of the 12 possible locations of the MTC has been covered once, and the difference of the toxicity probabilities between neighboring combinations are a mixture of 10% and 15%. The true orderings reflect various dominations of toxicity probabilities. More explicitly, scenario $R^{\left(1\right)}$ assumes drug A has the largest effect on toxicity, followed by drug B, and then drug C. Hence, for instance, ${\tilde{d}}_{2,2,2}$ is less toxic than ${\tilde{d}}_{2,3,1}$ because having a higher level of drug B has a larger effect on toxicity than higher level of drug C. Similarly, ${\tilde{d}}_{3,3,1}$ is more toxic than any of ${\tilde{d}}_{2,4,1},{\tilde{d}}_{2,2,2},{\tilde{d}}_{2,3,2},{\tilde{d}}_{2,4,2}$ because having a higher level of drug A is the most effective on toxicity. The dominations of each scenario are assumed as follows.

$$\begin{array}{ll} & R^{\left(1\right)}:A\to B\to C;\;R^{\left(2\right)}:A\to C\to B;\;R^{\left(3\right)}:B\to A\to C;\\ & R^{\left(4\right)}:B\to C\to A;\;R^{\left(5\right)}:C\to A\to B;\;R^{\left(6\right)}:C\to B\to A; \end{array}$$

$$\begin{array}{ll} & R^{\left(7\right)}:A\to (\text{alternate between}\;B,C);\\ & R^{\left(8\right)}:(\text{alternate between}\;C,B)\to A;\\ & R^{\left(9\right)}:B\to (\text{alternate between}\;A,C);\\ & R^{\left(10\right)}:(\text{alternate between}\;C,A)\to B;\\ & R^{\left(11\right)}:C\to (\text{alternate between}\;A,B);\\ & R^{\left(12\right)}:(\text{alternate between}\;C,A)\to C. \end{array}$$

**TABLE 3:** Toxicity scenarios.

				Drug A			Drug A			Drug A		
				$a_{1}$	$a_{2}$	$a_{3}$	$a_{1}$	$a_{2}$	$a_{3}$	$a_{1}$	$a_{2}$	$a_{3}$
				Scenario $R^{\left(1\right)}$			Scenario $R^{\left(2\right)}$			Scenario $R^{\left(3\right)}$		
Drug C	$c_{2}$		$b_{4}$		**0.25**	0.70		0.50	0.90		0.70	0.90
		Drug	$b_{3}$		0.10	0.50		0.40	0.80		**0.25**	0.50
		B	$b_{2}$		0.03			**0.25**			0.10	
			$b_{1}$									
	$c_{1}$		$b_{4}$		0.15	0.60		0.15	0.70		0.60	0.80
		Drug	$b_{3}$		0.05	0.40		0.10	0.60		0.15	0.40
		B	$b_{2}$	0.01	0.01		0.03	0.05		0.03	0.05	
			$b_{1}$	0.01			0.01			0.01		
				Scenario $R^{\left(4\right)}$			Scenario $R^{\left(5\right)}$			Scenario $R^{\left(6\right)}$		
Drug C	$c_{2}$		$b_{4}$		0.65	0.75		0.70	0.90		0.75	0.85
		Drug	$b_{3}$		0.15	**0.25**		0.60	0.80		0.55	0.65
		B	$b_{2}$		0.03			0.50			0.40	
			$b_{1}$									
	$c_{1}$		$b_{4}$		0.40	0.55		0.15	0.40		0.15	**0.25**
		Drug	$b_{3}$		0.05	0.10		0.10	**0.25**		0.05	0.10
		B	$b_{2}$	0.01	0.01		0.03	0.05		0.01	0.03	
			$b_{1}$	0.01			0.01			0.01		
				Scenario $R^{\left(7\right)}$			Scenario $R^{\left(8\right)}$			Scenario $R^{\left(9\right)}$		
Drug C	$c_{2}$		$b_{4}$		0.60	0.95		0.75	0.85		0.15	**0.25**
		Drug	$b_{3}$		0.50	0.80		0.55	0.65		0.01	0.03
		B	$b_{2}$		0.15			0.05			0.01	
			$b_{1}$									
			$b_{4}$		0.40	0.90		**0.25**	0.40		0.05	0.10
	$c_{1}$	Drug	$b_{3}$		**0.25**	0.70		0.10	0.15		0.01	0.01
		B	$b_{2}$	0.05	0.10		0.01	0.03		0.01	0.01	
			$b_{1}$	0.01			0.01			0.01		
				Scenario $R^{\left(10\right)}$			Scenario $R^{\left(11\right)}$			Scenario $R^{\left(12\right)}$		
Drug C	$c_{2}$		$b_{4}$		0.85	0.99		0.85	0.90		0.70	0.90
		Drug	$b_{3}$		0.80	0.95		0.75	0.80		0.65	0.80
		B	$b_{2}$		0.70			0.70			0.40	
			$b_{1}$									
	$c_{1}$		$b_{4}$		0.65	0.90		0.55	0.65		0.60	0.85
		Drug	$b_{3}$		0.60	0.75		0.50	0.60		0.50	0.75
		B	$b_{2}$	0.40	0.50		**0.25**	0.40		0.10	**0.25**	
			$b_{1}$	**0.25**			0.10			0.01		

### 5.3 Ordering Specification

**FIGURE 3:**
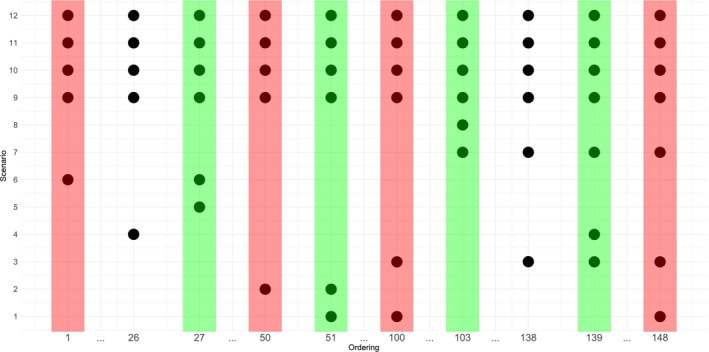
Correct ordering groups under the 12 scenarios in Table 3.

Given the 12 scenarios in Table 3, we look for the smallest number of orderings that cover the correct ordering group under each of these scenarios. Figure 3 gives the correct ordering group under selected orderings. The y‐axis plots the 12 scenarios, and the x‐axis shows the orderings. A dot at coordinate (m,r) means ordering ${𝒪}_{m}$ belongs to the correct group under scenario $R^{\left(r\right)}$, m=1,…,148, r=1,…,12. For example, the first column tells that ordering 1 belongs to the correct group under scenarios 6, 9, 10, 11, 12. The second column implies that ordering 26 (orderings 2‐25 in between are omitted, the full figure with 148 orderings can be found in the Supporting Information) is consistent under orderings 4, 9, 10, 11, 12. To fulfil Theorem 1, columns should be selected such that each row contains at least one dot. For example, ordering 27, 51, 103, 139 (highlighted in green) satisfies this condition. On the other hand, orderings 1, 50, 100, 148 (highlighted in red) does not satisfy the requirement as the correct group under scenarios 5 and 8 are not covered.

In the simulation study, the following choices of orderings will be compared. The last choice, inconsistent 4 orderings, is included to demonstrate the importance of using consistent orderings.

- All 148 orderings.
- Scenario‐specific 4 orderings: ordering 27, 51, 103, 139.
- Scenario‐agnostic 8 orderings from the full list: selected from the full list of 148 orderings in Section 4.2, ordering 2, 15, 29, 49, 73, 116, 134, 148.
- Scenario‐agnostic orderings from the Adding‐Refining algorithm: run the algorithm 10 times gives the following choices of orderings in Table 4. All of them cover the correct group under all 48 order‐scenarios, as shown in the Supporting Information.

**TABLE 4:** 10 choices of orderings from the Adding‐Refining algorithm.

	Orderings									
(1)	2	18	23	30	44	68	100	127	133	
(2)	1	17	29	51	92	93	114	118	143	
(3)	1	26	32	38	51	88	108	120	121	143
(4)	1	12	31	48	71	99	115	138		
(5)	8	24	28	51	91	108	110	118	141	
(6)	1	25	36	49	70	87	104	126	132	144
(7)	1	19	34	52	70	91	107	145		
(8)	1	17	33	35	38	64	84	106	139	142
(9)	2	9	25	27	52	69	106	134	146	
(10)	2	16	35	51	86	100	115	117	142	

*Note:* Inconsistent 4 orderings: ordering 1, 50, 100, 148, which is shown to be inconsistent from Figure 3.

Note that the all 148 orderings option is normally not viable in lots of real trials, where the grid will be much larger, and it is impossible to list out all orderings. In such cases, the use of the Adding‐Refining algorithm becomes crucial for both the scenario‐specific and scenario‐agnostic specification options. The Adding step would provide the full list of order‐scenarios and a list of potential orderings to be refined. Then, depending on the specification, the list of potential orderings can either be refined on the full order‐scenarios or a smaller number specified scenarios, both can be achieved via the Refining step. In Section S6 of the Supporting Information, an additional simulation study has been done on a 4×2×4 grid under the setting of the second motivating example. This gives an example where listing all orderings is impossible, but it is relatively easy and fast to list out all 6118 order‐scenarios.

### 5.4 Results

**FIGURE 4:**
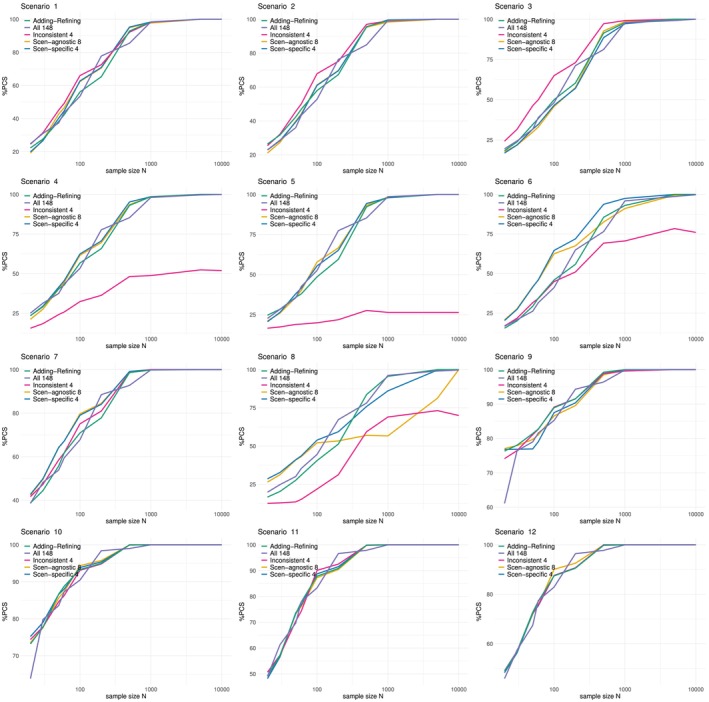
%PCS vs. sample size for 5 specifications of orderings. Each sub‐plot corresponds to one scenario, colors corresponds to ordering specification methods. All estimations based on $10^{4}$ simulations.

The PCS against sample sizes are plotted in Figure 4, where each sub‐plot corresponds to a scenario, and the 5 ordering specification methods are shown by colors. The four consistent choices of orderings (shown in green, purple, yellow, and blue) all have the PCS converges to 100% under all 12 scenarios, as expected. The PCS are very similar under these four consistent choices except under scenario 8, where the PCS under the scenario‐agnostic 8 orderings remains low until sample size N=1000.

On the other hand, the inconsistent 4 orderings (shown in pink) does not lead to a consistent design under scenarios 4, 5, 6, 8. From Figure 3, it can be seen that the correct ordering groups under scenario 4, 5, 8 are not covered by the inconsistent 4 orderings highlighted in red, and thus the inconsistencies of the design are as expected. Under scenario 6, ordering 1 belongs to the correct group, and the PCS does eventually converge to 100% when the sample size is increased to $N=5\times 10^{6}$. The reason for requiring a much larger sample size to converge is that under all the other three incorrect orderings, ordering 50, 100, 148, the MTC ${\tilde{d}}_{3,4,1}$ is labeled $d_{11}$, which makes it particularly hard to reach.

Apart from the asymptotic convergence, the PCS at small sample sizes is also of practical interest. Table 5a compares the 5 ordering specifications under N=60 patients. The values for Adding‐Refining is the average of the 10 outputs from the algorithm. The four consistent choices (first 4 rows) all give the (geometric) mean PCS under the 12 scenarios higher than 50%, whereas the inconsistent 4 orderings has mean PCS 45%. This shows that the consistency, although being an asymptotic property, can also affect the efficiency at small sample sizes. In particular, under scenarios 4, 5, 8, when the correct ordering group has not been covered by the inconsistent 4 ordering, its PCS are particularly low.

**TABLE 5:** PCS at sample size N=60 under 5 choice of ordering specifications.

	Scenario												
	1	2	3	4	5	6	7	8	9	10	11	12	Mean
(a) Equal prior probabilities of the orderings included													
Scen‐specific 4	42.4	41.5	33.0	48.4	37.9	48.2	69.9	44.5	80.2	87.3	78.3	76.2	54.4
All 148	45.1	40.3	39.5	43.6	42.0	33.8	61.6	35.9	82.2	88.5	79.7	75.6	52.4
Scen‐agnostic 8	46.9	38.5	44.5	43.5	42.9	37.1	62.5	26.0	81.2	87.8	75.4	76.8	51.8
Adding‐Refining	42.4	47.0	38.4	42.8	38.0	34.5	61.8	31.1	82.8	88.8	77.9	77.2	51.7
Inconsistent 4	49.2	47.9	51.3	26.3	18.0	35.0	59.9	14.0	81.1	88.4	79.7	73.6	45.1
(b) n.consis prior probabilities of orderings													
Scen‐specific 4	44.6	43.8	36.9	49.9	41.0	47.0	68.0	43.6	81.5	88.4	78.1	76.9	55.7
All 148	46.8	42.0	45.6	42.8	38.4	34.0	58.4	39.6	83.6	88.0	77.6	75.2	53.1
Scen‐agnostic 8	46.3	41.3	44.2	46.5	38.1	35.8	65.0	31.6	82.6	87.8	78.8	76.9	52.9
Adding‐Refining	45.3	43.5	46.7	39.3	43.1	33.7	59.0	28.2	82.7	89.1	78.2	76.1	52.1
Inconsistent 4	50.5	52.4	56.1	22.9	22.7	39.1	62.5	15.4	81.5	87.1	77.8	78.6	47.3

*Note:* All estimates based on $10^{4}$ simulations.

Among the four consistent choices of orderings, the scenario‐specific 4 orderings gives the highest mean PCS, followed by all 148 orderings. In general, [16] shown that there are two elements highly associated with the PCS at small sample sizes, the number of orderings, and the number of orderings in the correct group. Including less orderings would generally lead to better PCS, exemplified by the highest PCS under the scenario‐specific 4 orderings. The scenario‐agnostic 8 orderings gives a slightly higher PCS than the average output from the Adding‐Refining algorithm, which contains a mixture of 8, 9, and 10 orderings. Furthermore, having more orderings from the correct group would also help the PCS at small sample sizes, which explains the second highest PCS under all 148 orderings.

## 6 Prior Probability of Orderings

One observation from Table 5a is that, when the number of orderings included is small, the POCRM can have particularly small PCS under some scenarios, although the mean PCS is high. This is exemplified by the scenario‐specific 4 orderings under scenario 3, where it has PCS 33%, whereas, under all 148 orderings, the PCS is 40%, or under the scenario‐agnostic 8 orderings, the PCS is 45%. The reason is that under the scenario‐specific 4 orderings, only one ordering, ordering 139, from the correct group has been included. On the other hand, under the scenario‐agnostic 8 orderings, 2 from the correct group, ordering 134 and 148, are included. Under all 148 orderings, all 32 orderings in the correct group are included.

One possibility to mitigate this is to adjust the prior probabilities of orderings. Conventionally, equal prior probabilities for all orderings included are used to reflect the fact that, no information is available about which ordering is preferable than the others. As discussed in Section 3, the prior probabilities of orderings do not affect the consistency of the design as long as no ordering receive zero prior probability. Nevertheless, they may affect the efficiency at small sample sizes. In particular, motivated from the observation above, it seems that having more orderings in the correct group may help the efficiency. Hence, orderings that belongs to more correct groups should receive higher prior probability.

Formally, the metric n.consis tells the number of correct group an ordering belongs to.

$$\begin{array}{ll} \text{n.consis}(m) & =\overset{R}{\underset{r=1}{\sum }}1\{{𝒪}_{m}\in \text{correct ordering group}\text{under scenario}\;R^{\left(r\right)}\}, \end{array}$$

where m=1,…,M are the orderings included, and $R^{\left(r\right)}$, r=1,…,R, are scenarios being considered. Then, the prior probabilities of orderings are proportional to n.consis.

$$p(m)\propto n.\text{consis}(m),\;\underset{m}{\sum }p(m)=1.$$

This is referred to as the n.consis prior.

For example, among the 12 scenarios specified in Table 3, ordering 1 belongs to the correct group under scenario 6, 9, 10, 11, 12, and thus n.consis (1)=5. Graphically, n.consis (m) corresponds to the number of dots in the column of ordering ${𝒪}_{m}$ in Figure 3. Hence, it can be read from the plot that the scenario‐specific 4 orderings have n.consis(27)=n.consis(51)=n.consis(103)=6, n.consis(139)=7. The prior probabilities are therefore p(27)=p(51)=p(103)=6/25=0.24, p(139)=7/25=0.28. This can be similarly applied to other specifications of orderings, which are summarized in Table 6.

**TABLE 6:** Prior probabilities of orderings under 4 specifications.

Scenario‐specific 4 orderings									
m	27	51	103	139					
p(m)	0.24	0.24	0.24	0.28					
Scenario‐agnostic 8 orderings									
m	2	15	29	49	72	116	134	148	
p(m)	0.116	0.116	0.116	0.116	0.093	0.140	0.140	0.163	
Adding‐Refining choice (1)									
m	2	18	23	30	44	68	100	127	133
p(m)	0.109	0.109	0.087	0.109	0.109	0.109	0.130	0.109	0.130
Inconsistent 4 orderings									
m	1	50	100	148					
p(m)	0.217	0.217	0.261	0.304					

Prior orderings for the other 9 outputs of the Adding‐Refining algorithm, and all 148 orderings are detailed in the Supporting Information. Table 5b gives the PCS at N=60 under n.consis prior. Comparing to equal prior in Table 5a, the mean PCS are higher under the n.consis prior under all choices of orderings. The magnitude of increase ranges from 2.2% under the inconsistent 4 orderings to 0.4% under the mean of 10 outputs of the Adding‐Refining algorithm. As expected, adjusting the prior probabilities of orderings has a larger impact when the number of orderings included is small. The increases in the mean PCS are more than 1% for the scenario‐specific 4 orderings, scenario‐agnostic 8 orderings, and inconsistent 4 orderings. Whereas, when the number of orderings are large, the impact of adjusting the prior is minimal. The changes in PCS are around 0.5% under all 148 orderings or the average of 10 Adding‐Refining outputs.

Furthermore, note that adjusting the prior weight of orderings helps can improve the PCS under a number of scenarios. For example, scenario 3 under the scenario‐specific 4 orderings is hard, since only 1 ordering, ordering 139, from the correct group has been included. Nevertheless, by giving ordering 139 a higher prior probability, from 0.25 to 0.28, the PCS has increased by 3.9%. However, if no ordering from the correct group is included, adjusting the prior does not translate into the higher PCS. Under the inconsistent 4 ordering, the PCS under scenarios 4, 5, 8 are still less than half of the other choices of orderings.

## 7 Conclusion

This article discusses the ordering specification of the POCRM under complex settings, using the combination of three drugs as an example. The orderings are specified based on the asymptotic consistency of the design, and a novel Adding‐Refining algorithm has been proposed to solve the practical problem that it can be infeasible to list out all possible orderings. Simulations studies show that with a small number of orderings, 4 for scenario‐specific and 8 for scenario‐agnostic, the POCRM design can achieve both asymptotic and finite sample performances as good as with all 148 orderings. Additional simulation studies have been done under $10^{4}$ randomly generated scenarios as well as 148 structured scenarios that cover all possible orderings (details in the Supporting Information). The good performances of the consistency‐based ordering specification approaches remain true under those scenarios.

It is recommended that the Adding‐Refining algorithm should always be used for two reasons. Firstly, as shown in Table 2, full listing becomes computationally impossible very quickly for two‐dimensional problems with ≥4 dose levels or three‐dimensional problems with ≥3 levels. Secondly, even if full listing is possible, comparisons shown in Table 4 of the main text show that the difference in operating characteristics between the two approaches is very limited, whereas Adding‐Refining is much more computationally efficient.

The prior probability of orderings are conventionally set to equal for all orderings being included into the POCRM. This article proposed a novel way to assign prior ordering probabilities based on asymptotic consistency. This has shown to increase the average PCS by 1‐2%. Nevertheless, the proposed method is scenario‐specific. In practical use, if the assumed scenarios are deemed reliable, We recommend using the n.consis option, as it offers on average around 2% higher PCS. On the other hand, if very little knowledge about the possible scenarios is available, a more general scenario‐agnostic method to assign prior ordering probabilities is a topic worth further investigating. Nevertheless, additional simulation results in the Supporting Information show that, when the prior weights are obtained from misspecified scenarios, the n.consis weights would not deteriorate the model performance.

An alternative approach to deal with high‐dimensional settings is the local POCRM [22]. The POCRM is implemented locally in the adjacent region of the current dose combination, forming a so‐called local space containing a smaller number of treatment regimens and orderings. One potential future work would be to study the asymptotic properties of the local POCRM and compare with the method proposed in this article.

Additionally, an intuitively appealing approach to deal with uncertainties in orderings is to specify a path that connects the lowest and highest combinations, which contains a subset of the combinations whose orderings are known. Then, the CRM can be applied on this path. To avoid potential bias from choosing the path, several paths can be selected and the average result is taken. A comparison of this approach to the POCRM is detailed in the Supporting Information, and simulation results show that the POCRM, even under inconsistent orderings, has much better operating characteristics than this intuitive CRM‐on‐subpath approach. There are two reasons for the worse performance. Firstly, a pre‐specified path that does not contain the MTC is worse than an inconsistent ordering, because the latter can still assign patients to the MTC. Secondly, when combining the orderings/paths, the POCRM selects the most compatible ordering, and thus the inconsistent orderings will be largely down‐weighted. Conversely, the CRM‐on‐subpath method simply averages over all pre‐specified paths without any selection procedure, and whence the paths that do not include the MTC will make the average PCS much lower.

Finally, in recent Phase I clinical trials, focus has been shifted from identifying a single MTC to identifying a set of potential MTCs. One such example is described in Mozgunov et al. [19]. The POCRM, as a model‐based design, is exactly well‐suited for selecting multiple MTCs. This is because model‐based designs fits the combination‐toxicity relationship across the entire combination space. Thus, the safety review committee (SRC) receives estimated toxicity probabilities (and credible intervals) for all combinations. Given this information, the SRC has the flexibility to recommend which combination to give to the next cohort of patient, or recommend multiple MTCs for subsequent trial phases if needed. This is opposed to the rule‐based [23] or model‐assisted [8] designs, which typically only provide a single escalation/de‐escalation decision after each cohort and usually yield only one final recommended MTC. The simulations in our manuscript assign the next cohort of patients to the safe combination with the maximum toxicity probability, that is, the MTC. However, in reality, the SRC can overrule this decision and recommend another combination, as long as the combination is safe. Mozgunov et al. [19] demonstrated that the operational characteristics of the POCRM remain similar with or without overrules.

## Funding

This work was supported by Gates Cambridge Trust, National Institute for Health Research (Grant No. NIHR300576), Medical Research Council (Grant No. MC UU 00002/19), and Massey Comprehensive Cancer Center.

## Conflicts of Interest

The authors declare no conflicts of interest.

## Supporting information




**Data S1**: sim70345‐sup‐0001‐Supinfo.pdf.

## Data Availability

The authors have nothing to report.
