# Supplementary material for: Applying the Partial Order Continual Reassessment Method to High‐Dimensional Treatment Combinations
Source: Stat Med. 2026 Feb 27;45(6-7):e70345. doi: 10.1002/sim.70345 (PMC12948264; doi:10.1002/sim.70345)
Supplement: Supplementary file 1 — Data S1: sim70345‐sup‐0001‐Supinfo.pdf. [file SIM-45-0-s001.pdf]

Received: Added at production | Revised: Added at production | Accepted: Added at production

DOI: xxx/xxxx

SUPPLEMENTARY MATERIALS

Applying the partial order continual reassessment method to high-dimensional treatment combinations

1 | ORDERINGS

The 148 possible orderings are presented in Table S1 below. The numbers 1-12 correspond to combinations $\tilde{d}_{1,1,1}$, $\tilde{d}_{1,2,1}$, $\tilde{d}_{2,2,1}$, $\tilde{d}_{2,3,1}$, $\tilde{d}_{3,3,1}$, $\tilde{d}_{2,4,1}$, $\tilde{d}_{3,4,1}$, $\tilde{d}_{2,2,2}$, $\tilde{d}_{2,3,2}$, $\tilde{d}_{3,3,2}$, $\tilde{d}_{2,4,2}$, $\tilde{d}_{3,4,2}$, respectively.

Orderings													Orderings												
1	1	2	3	4	5	6	7	8	9	10	11	12	2	1	2	3	4	5	6	7	8	9	11	10	12
3	1	2	3	4	5	6	7	8	11	9	10	12	4	1	2	3	4	5	6	7	8	11	10	9	12
5	1	2	3	4	5	6	7	8	10	11	9	12	6	1	2	3	4	5	6	7	8	10	9	11	12
7	1	2	3	4	5	6	7	9	8	11	10	12	8	1	2	3	4	5	6	7	9	8	10	11	12
9	1	2	3	4	5	6	8	7	10	9	11	12	10	1	2	3	4	5	6	8	7	10	11	9	12
11	1	2	3	4	5	6	8	7	11	10	9	12	12	1	2	3	4	5	6	8	11	7	10	9	12
13	1	2	3	4	5	6	8	11	7	9	10	12	14	1	2	3	4	5	6	8	7	11	9	10	12
15	1	2	3	4	5	6	8	7	9	11	10	12	16	1	2	3	4	5	6	8	7	9	10	11	12
17	1	2	3	4	5	8	6	7	9	10	11	12	18	1	2	3	4	5	8	6	7	9	11	10	12
19	1	2	3	4	5	8	6	7	11	9	10	12	20	1	2	3	4	5	8	6	11	7	9	10	12
21	1	2	3	4	5	8	6	11	7	10	9	12	22	1	2	3	4	5	8	6	7	11	10	9	12
23	1	2	3	4	5	8	6	7	10	11	9	12	24	1	2	3	4	5	8	6	7	10	9	11	12
25	1	2	3	4	5	8	7	10	6	11	9	12	26	1	2	3	4	5	8	7	10	6	9	11	12
27	1	2	3	4	5	8	7	6	10	9	11	12	28	1	2	3	4	5	8	7	6	10	11	9	12
29	1	2	3	4	5	8	7	6	11	10	9	12	30	1	2	3	4	5	8	7	6	11	9	10	12
31	1	2	3	4	5	8	7	6	9	11	10	12	32	1	2	3	4	5	8	7	6	9	10	11	12
33	1	2	3	4	5	7	8	6	9	10	11	12	34	1	2	3	4	5	7	8	6	9	11	10	12
35	1	2	3	4	5	7	8	6	11	9	10	12	36	1	2	3	4	5	7	8	6	11	10	9	12
37	1	2	3	4	5	7	8	6	10	11	9	12	38	1	2	3	4	5	7	8	6	10	9	11	12
39	1	2	3	4	5	7	8	10	6	9	11	12	40	1	2	3	4	5	7	8	10	6	11	9	12
41	1	2	3	4	5	7	6	9	8	10	11	12	42	1	2	3	4	5	7	6	9	8	11	10	12
43	1	2	3	4	5	7	6	8	10	9	11	12	44	1	2	3	4	5	7	6	8	10	11	9	12
45	1	2	3	4	5	7	6	8	11	10	9	12	46	1	2	3	4	5	7	6	8	11	9	10	12
47	1	2	3	4	5	7	6	8	9	11	10	12	48	1	2	3	4	5	7	6	8	9	10	11	12
49	1	2	3	5	6	4	7	8	9	10	11	12	50	1	2	3	5	6	4	7	8	9	11	10	12
51	1	2	3	5	6	4	7	8	11	9	10	12	52	1	2	3	5	6	4	7	8	11	10	9	12
53	1	2	3	5	6	4	7	8	10	11	9	12	54	1	2	3	5	6	4	7	8	10	9	11	12
55	1	2	3	5	6	4	7	9	8	11	10	12	56	1	2	3	5	6	4	7	9	8	10	11	12
57	1	2	3	5	6	4	8	7	10	9	11	12	58	1	2	3	5	6	4	8	7	10	11	9	12
59	1	2	3	5	6	4	8	7	11	10	9	12	60	1	2	3	5	6	4	8	11	7	10	9	12

 © 2023 Copyright Holder Name

61	1	2	3	5	6	4	8	11	7	9	10	12	62	1	2	3	5	6	4	8	7	11	9	10	12
63	1	2	3	5	6	4	8	7	9	11	10	12	64	1	2	3	5	6	4	8	7	9	10	11	12
65	1	2	3	5	6	8	4	7	9	10	11	12	66	1	2	3	5	6	8	4	7	9	11	10	12
67	1	2	3	5	6	8	4	7	11	9	10	12	68	1	2	3	5	6	8	4	11	7	9	10	12
69	1	2	3	5	6	8	11	4	7	9	10	12	70	1	2	3	5	6	8	11	4	7	10	9	12
71	1	2	3	5	6	8	4	11	7	10	9	12	72	1	2	3	5	6	8	4	7	11	10	9	12
73	1	2	3	5	6	8	4	7	10	11	9	12	74	1	2	3	5	6	8	4	7	10	9	11	12
75	1	2	3	5	8	6	4	7	10	9	11	12	76	1	2	3	5	8	6	4	7	10	11	9	12
77	1	2	3	5	8	6	4	7	11	10	9	12	78	1	2	3	5	8	6	4	11	7	10	9	12
79	1	2	3	5	8	6	11	4	7	10	9	12	80	1	2	3	5	8	6	11	4	7	9	10	12
81	1	2	3	5	8	6	4	11	7	9	10	12	82	1	2	3	5	8	6	4	7	11	9	10	12
83	1	2	3	5	8	6	4	7	9	11	10	12	84	1	2	3	5	8	6	4	7	9	10	11	12
85	1	2	3	5	4	7	6	8	9	10	11	12	86	1	2	3	5	4	7	6	8	9	11	10	12
87	1	2	3	5	4	7	6	8	11	9	10	12	88	1	2	3	5	4	7	6	8	11	10	9	12
89	1	2	3	5	4	7	6	8	10	11	9	12	90	1	2	3	5	4	7	6	8	10	9	11	12
91	1	2	3	5	4	7	6	9	8	11	10	12	92	1	2	3	5	4	7	6	9	8	10	11	12
93	1	2	3	5	4	7	8	10	6	11	9	12	94	1	2	3	5	4	7	8	10	6	9	11	12
95	1	2	3	5	4	7	8	6	10	9	11	12	96	1	2	3	5	4	7	8	6	10	11	9	12
97	1	2	3	5	4	7	8	6	11	10	9	12	98	1	2	3	5	4	7	8	6	11	9	10	12
99	1	2	3	5	4	7	8	6	9	11	10	12	100	1	2	3	5	4	7	8	6	9	10	11	12
101	1	2	3	5	4	8	7	6	9	10	11	12	102	1	2	3	5	4	8	7	6	9	11	10	12
103	1	2	3	5	4	8	7	6	11	9	10	12	104	1	2	3	5	4	8	7	6	11	10	9	12
105	1	2	3	5	4	8	7	6	10	11	9	12	106	1	2	3	5	4	8	7	6	10	9	11	12
107	1	2	3	5	4	8	7	10	6	9	11	12	108	1	2	3	5	4	8	7	10	6	11	9	12
109	1	2	3	5	8	4	7	10	6	11	9	12	110	1	2	3	5	8	4	7	10	6	9	11	12
111	1	2	3	5	8	4	7	6	10	9	11	12	112	1	2	3	5	8	4	7	6	10	11	9	12
113	1	2	3	5	8	4	7	6	11	10	9	12	114	1	2	3	5	8	4	7	6	11	9	10	12
115	1	2	3	5	8	4	7	6	9	11	10	12	116	1	2	3	5	8	4	7	6	9	10	11	12
117	1	2	3	5	8	4	6	7	9	10	11	12	118	1	2	3	5	8	4	6	7	9	11	10	12
119	1	2	3	5	8	4	6	7	11	9	10	12	120	1	2	3	5	8	4	6	11	7	9	10	12
121	1	2	3	5	8	4	6	11	7	10	9	12	122	1	2	3	5	8	4	6	7	11	10	9	12
123	1	2	3	5	8	4	6	7	10	11	9	12	124	1	2	3	5	8	4	6	7	10	9	11	12
125	1	2	3	5	4	8	6	7	10	9	11	12	126	1	2	3	5	4	8	6	7	10	11	9	12
127	1	2	3	5	4	8	6	7	11	10	9	12	128	1	2	3	5	4	8	6	11	7	10	9	12
129	1	2	3	5	4	8	6	11	7	9	10	12	130	1	2	3	5	4	8	6	7	11	9	10	12
131	1	2	3	5	4	8	6	7	9	11	10	12	132	1	2	3	5	4	8	6	7	9	10	11	12
133	1	2	3	5	4	6	8	7	9	10	11	12	134	1	2	3	5	4	6	8	7	9	11	10	12
135	1	2	3	5	4	6	8	7	11	9	10	12	136	1	2	3	5	4	6	8	11	7	9	10	12
137	1	2	3	5	4	6	8	11	7	10	9	12	138	1	2	3	5	4	6	8	7	11	10	9	12
139	1	2	3	5	4	6	8	7	10	11	9	12	140	1	2	3	5	4	6	8	7	10	9	11	12
141	1	2	3	5	4	6	7	9	8	10	11	12	142	1	2	3	5	4	6	7	9	8	11	10	12
143	1	2	3	5	4	6	7	8	10	9	11	12	144	1	2	3	5	4	6	7	8	10	11	9	12
145	1	2	3	5	4	6	7	8	11	10	9	12	146	1	2	3	5	4	6	7	8	11	9	10	12
147	1	2	3	5	4	6	7	8	9	11	10	12	148	1	2	3	5	4	6	7	8	9	10	11	12

TABLE S1 Possible orderings of the 12 combinations.

2 | ILLUSTRATION OF DEFINITIONS AND THEOREMS

The definitions of *relabelling*, *correct ordering group*, and *order-scenario* are illustrated in the following $2 \times 2 \times 2$ example. The true toxicity probability $\mathbf{R}$ is shown on the left of Table S2, and the labels $d = \mathfrak{L}(\tilde{d}; \mathbf{R})$ are shown on the right. The target toxicity level is 0.25, and thus the MTC is $\tilde{d}_{1,1,2} = (a_1, b_1, c_2)$ in this case. Upon relabelling per Definition 2, the toxicity probabilities increase from $d_1 \to d_2 \to \cdots \to d_8$.

The correct ordering group defined in Definition 3 requires two conditions. Firstly, the MTC has to be ordered at the correct place. In this example, the label of the MTC is $\mathfrak{L}(\tilde{d}_{1,1,2}; \mathbf{R}) = d_4$, and thus any ordering in the correct group must have $\tilde{d}_{1,1,2}$ at the 4th place. Secondly, all combinations less toxic than the MTC need to be ordered before the MTC. In this example, there are three combinations less toxic than 0.25: $\tilde{d}_{1,1,1}$, $\tilde{d}_{2,1,1}$, and $\tilde{d}_{1,2,1}$. Any ordering in the correct group must have these three combinations at the first 3 places, but the ordering amongst these three is not restricted.

In total, there are 12 orderings belonging to the correct group. These are illustrated by Figure S1. (Panel A) assigns each combination a colour, which are used to show the orderings in (Panel B). For example, the first ordering in (Panel B) is $\tilde{d}_{1,1,1} \to \tilde{d}_{2,1,1} \to \tilde{d}_{1,2,1} \to \tilde{d}_{1,1,2} \to \tilde{d}_{2,2,1} \to \tilde{d}_{2,1,2} \to \tilde{d}_{1,2,2} \to \tilde{d}_{2,2,2}$. The figure shows that all correct orderings have the MTC

				True toxicities $\mathbf{R}$		Labels $d = \mathfrak{L}(\tilde{d}; \mathbf{R})$	
				Drug A		Drug A	
				a_1	a_2	a_1	a_2
Drug C	c_2	Drug B	b_2	0.50	0.75	d_6	d_8
			b_1	**0.25**	0.60	$\mathbf{d_4}$	d_7
	c_1	Drug B	b_2	0.15	0.40	d_3	d_5
			b_1	0.05	0.10	d_1	d_2

TABLE S2 Left: true toxicity probabilities, where the target toxicity level of 0.25 is shown in **bold**. Right: labels of combinations after applying the relabelling technique defined in Definition 2.

(light green) ordered at the 4th place. The three combinations less toxic than the MTC (dark green, orange, and purple) are all at the first three places.

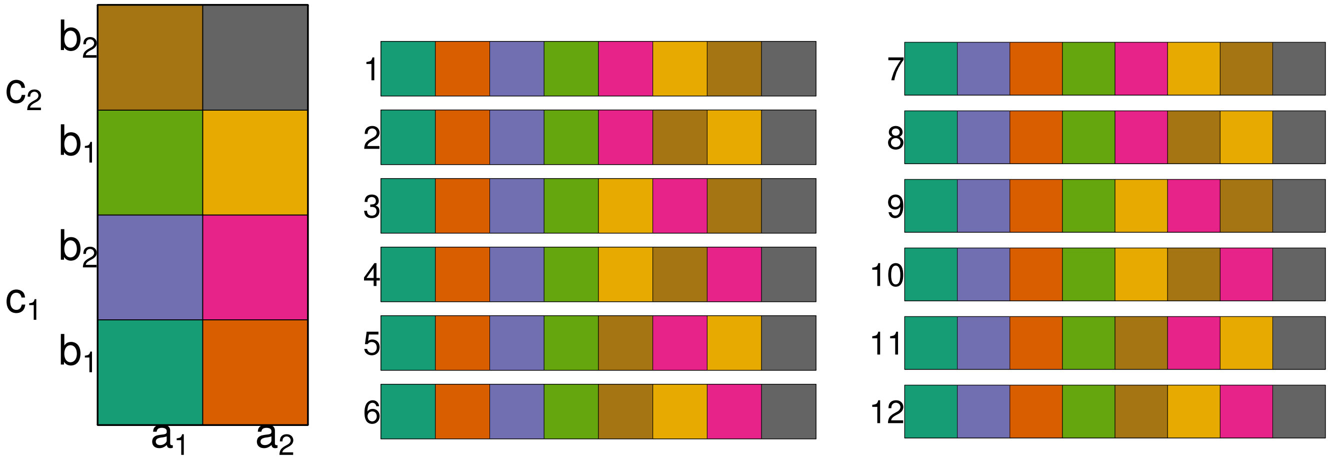

(b) 12 orderings in the correct ordering group.

(a) Each combination is assigned a colour.

FIGURE S1 Orderings in the correct ordering group.

The notion of order-scenario per Definition 4 is defined such that toxicity scenarios belonging to the same order-scenario lead to the same correct ordering group. As an example, scenarios $\mathbf{R}^{(1)}$ and $\mathbf{R}^{(2)}$ in Table S3 all belong to the same order-scenario as $\mathbf{R}$ in Table S2, whereas scenario $\mathbf{R}^{(3)}$ does not correspond to the same order-scenario. The differences between each scenario and $\mathbf{R}$ are highlighted in green. It can be seen that scenarios $\mathbf{R}^{(1)}$ and $\mathbf{R}^{(2)}$ correspond to orderings 7 and 5, respectively, in Figure S1 (Panel B), and thus still belong to the correct ordering group. On the other hand, scenario $\mathbf{R}^{(3)}$ has the MTC labelled as d_5 instead of d_4, and therefore its correct ordering does not belong to the same group.

				$\mathbf{R}^{(1)}$		$\mathbf{R}^{(2)}$		$\mathbf{R}^{(3)}$	
				Drug A		Drug A		Drug A	
				a_1	a_2	a_1	a_2	a_1	a_2
Drug C	c_2	Drug B	b_2	0.50	0.75	0.40	0.80	0.50	0.75
			b_1	**0.25**	0.60	**0.25**	0.60	**0.25**	0.60
	c_1	Drug B	b_2	0.15	0.40	0.15	0.50	0.15	0.20
			b_1	0.05	0.20	0.05	0.10	0.05	0.10

TABLE S3 Toxicity scenarios $\mathbf{R}^{(1)}, \mathbf{R}^{(2)}, \mathbf{R}^{(3)}$. The differences between each scenario with $\mathbf{R}$ in Table S2 are highlighted in green.

3 | FURTHER SIMULATION RESULTS

The root mean squared error (RMSE) between the true and estimated combination-toxicity relationships can be used as a metric to assess the goodness-of-fit of the design. Table S4 below shows the RMSE of the POCRM under 5 specifications of orderings (all 148, scenario-specific, scenario-agnostic, Adding-Refining, inconsistent) and 2 choices of prior weights under the 12 scenarios specified in the main text.

Overall, the mean RMSE under all ordering specifications are small. The mean difference between the true and fitted toxicity probabilities are around 10%, indicating a good fit of the true combination-toxicity relationships. The prior weights do not seem to affect the goodness-of-fit. Including all 148 orderings inflates the RMSE slightly by 3%. This is not surprising, since lots of orderings would have similar posterior probabilities, and thus the ordering selection step of the POCRM would randomly select an ordering. This leads to high variability of the orderings being selected, and thus deteriorates the overall RMSE.

Although the mean RMSE under the inconsistent 4 orderings is similar to those under consistent choices, the RMSEs are particularly large under scenarios 4 and 8. These are exactly the scenarios where no orderings in the correct ordering group has been included. This shows that using a small number of consistent orderings also helps the goodness-of-fit of the combination-toxicity relationships.

	Scenario												
	1	2	3	4	5	6	7	8	9	10	11	12	Mean
Scen-specific 4	0.09	0.08	0.10	0.07	0.13	0.11	0.15	0.11	0.06	0.08	0.10	0.11	0.10
All 148	0.12	0.17	0.17	0.13	0.18	0.17	0.19	0.17	0.05	0.08	0.09	0.11	0.13
Scen-agnostic 8	0.06	0.09	0.12	0.10	0.12	0.11	0.12	0.13	0.08	0.07	0.10	0.10	0.10
Adding-Refining	0.09	0.10	0.11	0.10	0.13	0.11	0.13	0.12	0.07	0.07	0.10	0.10	0.10
Inconsistent 4	0.05	0.09	0.15	0.19	0.14	0.08	0.09	0.17	0.08	0.07	0.11	0.09	0.10

(a) Equal prior probabilities of the orderings included.

	Scenario												
	1	2	3	4	5	6	7	8	9	10	11	12	Mean
Scen-specific 4	0.10	0.09	0.09	0.07	0.13	0.11	0.16	0.11	0.05	0.10	0.13	0.11	0.10
All 148	0.11	0.17	0.18	0.13	0.18	0.17	0.2	0.17	0.05	0.09	0.11	0.11	0.13
Scen-agnostic 8	0.06	0.10	0.12	0.10	0.12	0.12	0.10	0.14	0.09	0.08	0.13	0.07	0.10
Adding-Refining	0.09	0.10	0.11	0.09	0.13	0.11	0.13	0.12	0.07	0.08	0.12	0.09	0.10
Inconsistent 4	0.05	0.10	0.15	0.20	0.14	0.08	0.09	0.17	0.08	0.08	0.13	0.07	0.11

(b) `n.consis` prior probabilities of orderings.

TABLE S4 RMSE at sample size $N = 60$ under 5 choice of ordering specifications with equal prior weights (top) and weights defined based on `n.consis` (bottom). All estimates based on 10^4 simulations.

148 additional structured scenarios are added to further support the proposed methods, defined by sorting the monotonically increasing toxicity probabilities $(0.03, 0.05, 0.07, 0.10, 0.15, 0.25, 0.35, 0.45, 0.55, 0.65, 0.70, 0.75)$ according to the full list of 148 orderings. The simulation results at sample size $N = 60$, based on 10^4 simulations, are summarised in Table S5. Mean estimates and standard errors among the 148 scenarios are provided.

The following observations can be made from the simulation results:

- Scenario-agnostic vs. scenario-specific orderings: since the 4 scenario-specific orderings are specified based on the 12 scenarios used in the paper, they are not necessarily consistent under the 148 structured scenarios. Meanwhile, the scenario-agnostic 8 orderings ensures that the design is consistent under any scenario. This is reflected by the 4-5% lower PCS and the much higher standard errors. In particular, 58 out of the 148 structured scenarios have their correct ordering group uncovered by the 4 scenario-specific orderings. The PCS under these 58 scenarios is 22.1% with the now inconsistent 4 scenario-specific orderings compared to 28.0% with the consistent 8 scenario-agnostic orderings. The inconsistent 4 orderings remains inconsistent under the 148 structured scenarios, 90 out of the 148 scenarios have their correct ordering groups uncovered.
- Scenario-agnostic vs. Adding-Refining vs. all 148 orderings: these three choices of orderings are consistent regardless of scenarios. The PCS and RMSE under them are all very similar.
- Equal vs. `n.consis` prior weights: the `n.consis` prior weight is again scenario-dependent. Using the weights specified based on the 12 scenarios in the main text of the manuscript would not necessarily lead to improved operating characteristics

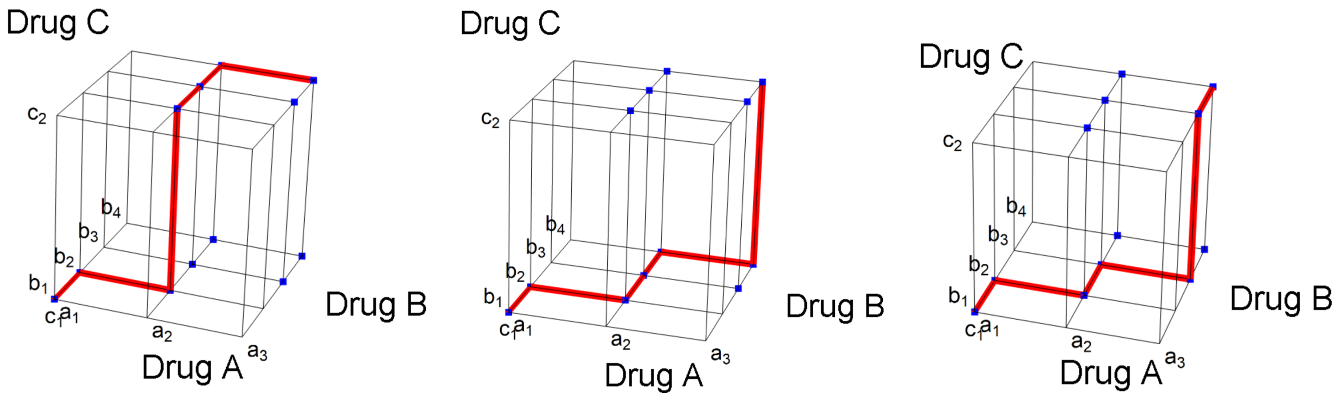

(a) Path 1 (b) Path 2 (c) Path 3

FIGURE S2 The 12 combinations are shown as blue dots, and the fixed paths are shown in red.

under the 148 structured or 10^4 random scenarios. Nevertheless, the difference between `n.consis` weight and equal weight are small, and very often within simulation error. This shows that even if the prior weights are elicited under misspecified scenarios, they would not seriously deteriorate the operating characteristics.

Orderings	Prior weight	%PCS (s.e.)	RMSE (s.e.)
All 148	equal	29.6 (3.5)	0.13 (0.003)
	`n.consis`	28.5 (4.6)	0.13 (0.003)
Scen-agnostic 8	equal	27.9 (4.8)	0.13 (0.004)
	`n.consis`	28.3 (3.6)	0.13 (0.005)
Adding-Refining	equal	28.1 (5.5)	0.13 (0.005)
	`n.consis`	27.7 (5.9)	0.13 (0.005)
Scen-specific 4	equal	24.4 (8.3)	0.14 (0.007)
	`n.consis`	25.0 (7.0)	0.14 (0.008)
Inconsistent 4	equal	23.5 (10.8)	0.13 (0.010)
	`n.consis`	22.4 (11.6)	0.13 (0.010)

TABLE S5 Mean PCS across 148 additional scenarios at sample size $N = 60$ under 5 ordering specifications and 2 prior weights. Consistent choices of orderings highlighted in green.

4 | CRM ON SUB-PATHS WITH KNOWN ORDERINGS

An alternative solution to unknown orderings is to apply CRM on a subset of combinations whose orderings are known. For example, The 3 paths on 7 combinations whose orderings are known are illustrated in Figure S2, and 10^4 simulations have been done under each path. The average result of these 3×10^4 simulations is being compared to the POCRM on the whole 12 combinations under 3×10^4 simulations. Let $\tilde{d}_{i,j,k} = (a_i, b_j, c_k)$ denote the combination with drug A at level a_i, $i = 1, \ldots, 3$, drug B at level b_j, $j = 1, \ldots, 4$, and drug C at level c_k, $k = 1, 2$. The three paths were chosen such that each combination is included in at least on path.

- Path 1: $\tilde{d}_{1,1,1} \to \tilde{d}_{1,2,1} \to \tilde{d}_{2,2,1} \to \tilde{d}_{2,2,2} \to \tilde{d}_{2,3,2} \to \tilde{d}_{2,4,2} \to \tilde{d}_{3,4,2}$.
- Path 2: $\tilde{d}_{1,1,1} \to \tilde{d}_{1,2,1} \to \tilde{d}_{2,2,1} \to \tilde{d}_{2,3,1} \to \tilde{d}_{2,4,1} \to \tilde{d}_{3,4,1} \to \tilde{d}_{3,4,2}$.
- Path 3: $\tilde{d}_{1,1,1} \to \tilde{d}_{1,2,1} \to \tilde{d}_{2,2,1} \to \tilde{d}_{2,3,1} \to \tilde{d}_{3,3,1} \to \tilde{d}_{3,3,2} \to \tilde{d}_{3,4,2}$.

The CRM[1] used in this simulation takes the parametric form

$$p_{i,j,k} = \alpha_{i,j,k}^{\exp(a)},$$

	Scenario												
	1	2	3	4	5	6	7	8	9	10	11	12	Mean
Scen-specific 4	42.4	41.5	33.0	48.4	37.9	48.2	69.9	44.5	80.2	87.3	78.3	76.2	54.4
All 148	45.1	40.3	39.5	43.6	42.0	33.8	61.6	35.9	82.2	88.5	79.7	75.6	52.4
Scen-agnostic 8	46.9	38.5	44.5	43.5	42.9	37.1	62.5	26.0	81.2	87.8	75.4	76.8	51.8
Adding-Refining	42.4	47.0	38.4	42.8	38.0	34.5	61.8	31.1	82.8	88.8	77.9	77.2	51.7
Inconsistent 4	49.2	47.9	51.3	26.3	18.0	35.0	59.9	14.0	81.1	88.4	79.7	73.6	45.1
CRM	22.3	24.5	20.9	21.0	21.0	15.2	48.7	20.8	60.8	79.2	73.4	78.5	33.7

TABLE S6 PCS under the POCRM on 12 combinations under 5 choices of orderings, and under the CRM with subsets of 7 combinations.

where $p_{i,j,k}$ is the toxicity probability of $\tilde{d}_{i,j,k}$. The values $\alpha_{i,j,k}$ makes a vector of monotonically increasing toxicity skeletons, which represent prior estimates of the toxicity probability at $\tilde{d}_{i,j,k}$. The model parameter $a \in \mathbb{R}$ follows a prior distribution $a \sim \mathcal{N}(0, \sigma^2)$. The prior variance $\sigma^2 = 1.34$ has been shown to yeild good operating characteristics[2], and calibrated skeletons were generated using the `getprior()` function from the `dfcrm` R package[3,4].

The simulations were conducted under the same 12 scenarios detailed in Table 2 of the main text, where $N = 60$ patients (20 cohorts of 3 patients) were assumed for the POCRM. As the CRM is applied to subsets of only 7 combinations, the sample size is reduced to $N = 36$ (12 cohorts of 3 patients) to maintain the same ratio between the number of patients and the number of combinations. The PCS under the CRM is compared to the POCRM with various choices of orderings, and the results are shown in Table S6 below. The first 4 rows correspond to the POCRM under 4 specifications of consistent orderings, and the 5th row corresponds to an inconsistent ordering. The results for the CRM are added to the last row.

Overall, the mean PCS for the CRM is approximately 10% lower than the POCRM with inconsistent orderings and 15% lower than the POCRM with consistent orderings. Individually, the 12 scenarios can be categorised into 3 categories based on the number of pre-specified paths that cover the MTC. The 4 green scenarios (scenario 9, 10, 11, 12) have MTCs included in all 3 paths for the CRM, and thus the CRM has PCS comparable (or slightly lower due to the smaller sample size) to that of the POCRM. Under the orange scenario (scenario 7), two out of three paths cover the MTC, and the PCS for the CRM is around 10% lower than the POCRM. Under the other 7 uncoloured scenarios (scenario 1, 2, 3, 4, 5, 6, 8), only one out of 3 paths covers the MTC, and the PCS under the CRM is roughly only half of that under the POCRM. In particular, under scenario 4 and 6, the PCS under the CRM is lower than the POCRM with inconsistent orderings.

The worse performance of the CRM is not surprising for two reasons. Firstly, a pre-specified path that does not cover the MTC is worse than an inconsistent ordering, because the latter can still assign patients to the MTC. Secondly, when combining the orderings/paths, the POCRM selects the most compatible ordering, and thus the inconsistent orderings will be largely down-weighted. Conversely, the proposed CRM method simply averages over all pre-specified paths without any selection procedure, and whence the paths that do not include the MTC will make the average PCS much lower.

5 RANDOM SCENARIOS

The proposed ordering specification approaches are also evaluated under 10^4 randomly generated scenarios of combination-toxicity relationships. The same set up as described in Section 4 in the main manuscript has been used.

The *pseudo-uniform* approach suggested in Clertant and O'Quigley (2017)[5] has been adopted to generate a vector of monotonically increasing toxicity probabilities $\boldsymbol{q}^s = (q_1^s, \ldots, q_L^s)$, $L = 12$. Explicitly, $\boldsymbol{q}^s$ is simulated using the following 3 steps.

1. Select the position of the MTC uniformly from $\{1. \ldots, L\}$, resulting in the MTC being the kth toxic combination.
2. Randomly generate an upper bound on the toxicity probabilities $B_s = \theta + (1 - \theta)M$, where $\theta = 0.25$ is the target toxicity level, and $M \sim \text{Beta}(\max\{L - k, 0.5\}, 1)$.
3. Uniformly simulate $k - 1$ ordered values from $(0, \theta)$, set as $(q_1^s, \ldots, q_{k-1}^s)$; simulate $12 - k$ values from (θ, B_s), set as $(q_{k+1}^s, \ldots, q_{12}^s)$. Set $q_k^s = \theta$.

The rationale for setting the upper bound B_s is to avoid having lots of extreme scenarios where all combinations are overly toxic, while still allowing such extreme scenarios to exist. Then, an ordering $\mathcal{O}_s$ is selected uniformly from all possible orderings. Sorting $\boldsymbol{q}$ according to $\mathcal{O}_s$ gives random scenario $\mathbf{R}^{(s)}$, for $s = 1, \ldots, 10^4$. One simulation has been conducted under each of the 10^4 random scenarios, and the mean PCS, PAS (proportion of acceptable selection), and RMSE, together with their 95%

confidence intervals (C.I.s), are summarised in Table S7, where a combination is acceptable if its toxicity is between 20% and 30%. Note that since the randomly generated scenarios do not control the toxicity difference between neighbouring combinations, there can be several combinations with toxicity arbitrarily close to 25%. It is infeasible for the model to distinguish such combinations with limited sample size, and thus the PCS under random scenarios will inevitably be lower.

The PCS and PAS under random scenarios show a very similar pattern as the structured scenarios. All the observations under structured scenarios in Section 3 above remain true under random scenarios. Consistent choices of orderings give higher PCS and PAS than inconsistent choices, and the differences, although seemingly small, are generally significant with 95% confidence.

Orderings	Prior weight	%PCS (C.I.)	%PAS (C.I.)	RMSE
All 148	equal	28.9 (28.0, 29.8)	58.0 (57.0, 59.0)	0.14
	`n.consis`	28.8 (27.9, 29.7)	59.0 (58.0, 60.0)	0.13
Scen-agnostic 8	equal	28.4 (27.5, 29.3)	58.8 (57.8, 59.8)	0.13
	`n.consis`	28.9 (28.0, 29.8)	58.3 (57.3, 59.3)	0.14
Adding-Refining	equal	28.7 (27.8, 29.6)	58.9 (57.9, 59.9)	0.13
	`n.consis`	28.8 (27.9, 29.7)	59.4 (58.4, 60.4)	0.13
Scen-specific 4	equal	27.8 (26.9, 28.7)	56.8 (55.8, 57.8)	0.14
	`n.consis`	27.8 (26.9, 28.7)	56.3 (55.3, 57.3)	0.14
Inconsistent 4	equal	27.9 (27.0, 28.8)	57.2 (56.2, 58.2)	0.14
	`n.consis`	28.9 (28.0, 29.8)	57.7 (56.7, 58.7)	0.14

TABLE S7 Mean PCS and 95% C.I.s. across 10^4 random scenarios at sample size $N = 60$ under 5 ordering specifications and 2 prior weights. Consistent choices of orderings highlighted in green.

6 | ADDITIONAL SIMULATION STUDY: $4 \times 2 \times 4$ GRID

The example with 12 combinations and 148 orderings in the main text may make the use of the Adding-Refining algorithm appear redundant. To fully test the Adding-Refining algorithm, we have added another example with a $4 \times 2 \times 4$ grid, shown in Figure S3, where 4 levels of drug A and 2 levels of drug B are combined with 4 administration schedules. It is impossible to list out the at least more than 2 million orderings, whereas the “Adding" step of the Adding-Refining algorithm allows listing out the 6224 order-scenarios and 6118 candidate orderings that are consistent under all order-scenarios. Then, we can refine the 6118 candidate orderings either based on all of the 6224 order-scenarios to obtain 944 scenario-agnostic orderings, or based on a smaller number of specified scenarios to obtain 20 scenario-specific orderings (detailed below). Both can be achieved by the “Refining" step of the Adding-Refining algorithm.

To make the test thorough, the simulation is done under 140 scenarios such that all possible locations of the MTCs are considered. More explicitly, the scenarios are generated in the following steps.

1. Fix the level of drug B at b_1, which gives a 4×4 grid for drug A and schedules.
2. Consider all possible locations of the MTCs, and the toxicity differences between neighbouring combinations are 10-15%. This gives 70 scenarios shown in Figure S4.
3. Under the same levels of drug A, a_i and schedule s_k, increasing the level of drug B from b_1 to b_2 increases the toxicity by 10%, i.e. $d_{i,2,k}$ is 10% more toxic than $d_{i,1,k}$.
4. Exchange the roles of b_1 and b_2 gives 70 more scenarios

The target toxicity level is set to 30%, a combination is *acceptable* if its toxicity is within $[20\%, 35\%]$ and is over-toxic if higher than 35%. The sample size is fixed at $N = 48$, enrolling patients in 16 cohorts of 3.

The scenario-specific approach is based on the 32 scenarios (16 for drug B fixed at b_1 and another 16 for drug B at b_2) illustrated in Figure S4a with 1 MTC on the 4×4 drug A-schedule grid. Consistency on these 32 scenarios requires 20 orderings (listed in the Supplementary Materials). For comparison, we also randomly draw 20 orderings from the 6118 candidate orderings, which do not cover the correct ordering groups under all 32 scenarios.

We compare the PCS (proportion of correct selection of the MTC), PAS (proportion of acceptable selection), PToxS (proportion of over-toxic selection), and RMSE (root mean squared error) under the scenario-agnostic 944, scenario-specific

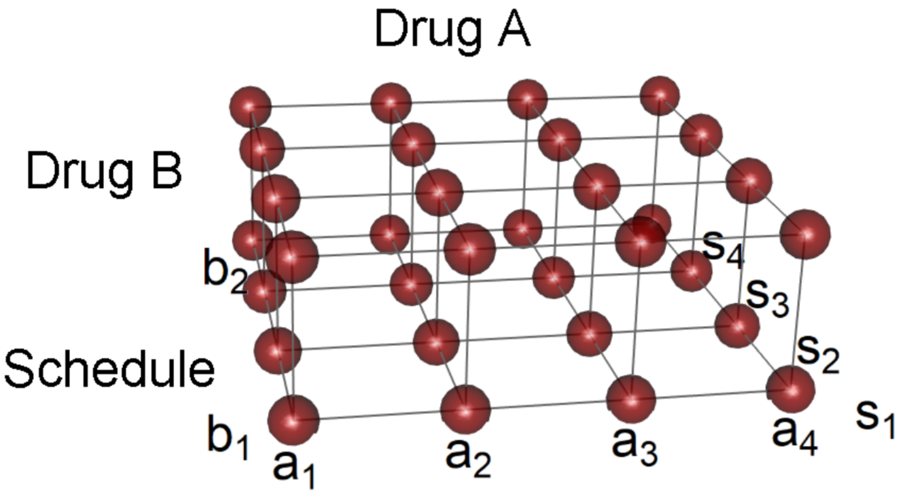

FIGURE S3 32 combinations on the $4 \times 2 \times 4$ grid. Combinations shown in red dots. 4 levels of drug A on the x (left-right) axis, 2 levels of drug B on the z (vertical) axis, and 4 levels of schedule on the y (front-back) axis.

20, and inconsistent 20 orderings. The results are shown in Table S8 below. The prior probability of orderings are set to equal, and all estimates based on 10^4 simulations. It can be seen that the conclusion aligns with the example shown in the main text with 12 combinations. Both the scenario-agnostic and the scenario-specific orderings have PCS 5-8% and PAS 5-15% higher than the inconsistent orderings. The inconsistent orderings also has the highest over-toxic selection among the three ordering specifications, while the RMSE is similar among all orderings. The 20 scenario-specific ordering specification has better operating characteristic than the 944 scenario-agnostic orderings. This gives another example that, when both orderings are consistent, including a large number of orderings could deteriorate the operating characteristics.

	PCS	(s.e.)	PAS	(s.e.)	PToxS*	(s.e.)	RMSE	(s.e.)
Scen-agnostic	33.53	10.15	65.00	9.41	17.99	11.76	0.16	0.01
Scen-specific	36.85	13.18	78.06	9.12	13.99	5.69	0.16	0.01
Inconsistent	28.19	14.50	60.99	11.69	21.00	9.00	0.16	0.01

TABLE S8 Geometric mean over 140 scenario for the $4 \times 2 \times 4$ grids under the POCRM with 3 specifications of orderings. (∗) scenarios with no overly toxic combination is excluded from the geometric mean. All estimates based on 10^4 simulations.

REFERENCES

1. O'Quigley J, Pepe M, Fisher L. Continual Reassessment Method: A Practical Design for Phase 1 Clinical Trials in Cancer. *Biometrics.* 1990;46(1):33–48.
2. O'Quigley J, Shen L. Continual Reassessment Method: A Likelihood Approach. *Biometrics.* 1996;52(2):673-84.
3. Lee S, Cheung K. Model Calibration in the Continual Reassessment Method. *Clinical Trials.* 2009;6:227-238.
4. Cheung K. Dose-Finding by the Continual Reassessment Method. 2013.
5. Clertant M, O'Quigley J. Semiparametric dose finding methods. *Journal of the Royal Statistical Society. Series B, Statistical methodology.* 2017;79(5):1487-1508.

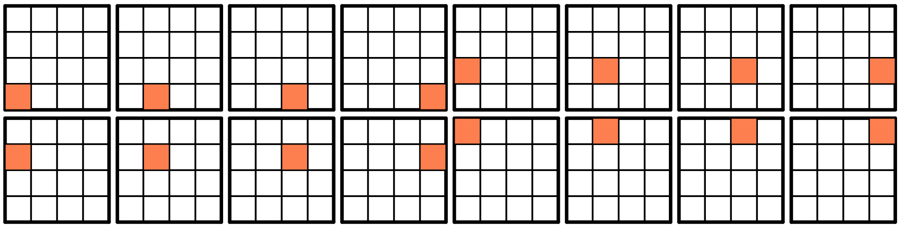

(a) 16 scenarios with 1 MTC.

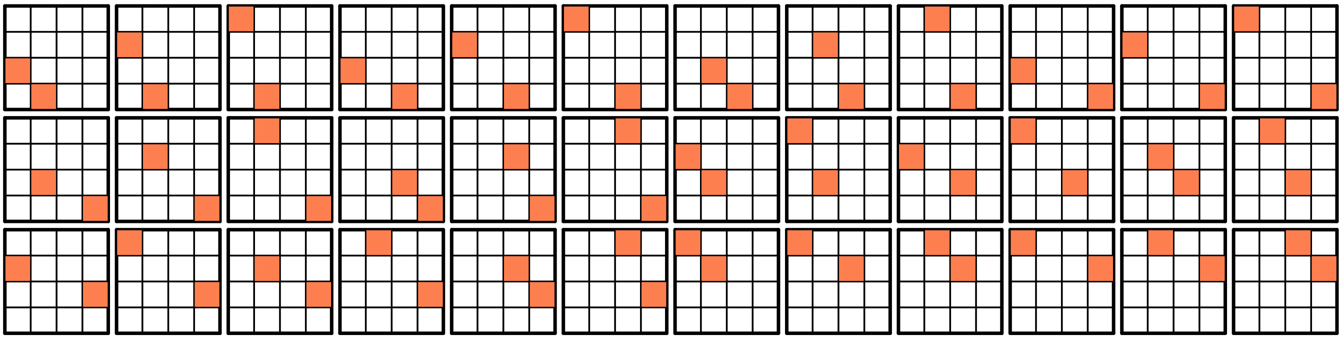

(b) 36 scenarios with 2 MTCs.

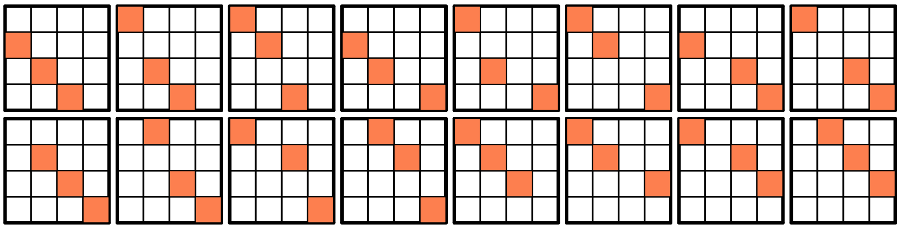

(c) 16 scenarios with 3 MTCs.

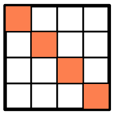

(d) 1 scenario with 4 MTCs.

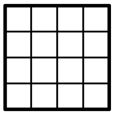

(e) 1 scenario with no MTC.

FIGURE S4 Locations of MTCs under the 4×4 grid for drug A and schedule when fixing the level of drug B. MTCs shown in red.
